# A novel phosphoproteomic landscape evoked in response to type I interferon in the brain and in glial cells

**DOI:** 10.1186/s12974-021-02277-x

**Published:** 2021-10-16

**Authors:** Barney Viengkhou, Melanie Y. White, Stuart J. Cordwell, Iain L. Campbell, Markus J. Hofer

**Affiliations:** 1 School of Life and Environmental Sciences, Charles Perkins Centre and Sydney Institute for Infectious Diseases, The University of Sydney, Sydney, NSW 2006 Australia; 2 School of Life and Environmental Sciences, School of Medical Sciences, Charles Perkins Centre and Sydney Mass Spectrometry, The University of Sydney, Sydney, NSW 2006 Australia

**Keywords:** Interferon, Microglia, Astrocyte, Phosphoproteomics, Cerebral type I interferonopathy, Neurodegenerative disease

## Abstract

**Background:**

Type I interferons (IFN-I) are key responders to central nervous system infection and injury and are also increased in common neurodegenerative diseases. Their effects are primarily mediated via transcriptional regulation of several hundred interferon-regulated genes. In addition, IFN-I activate several kinases including members of the MAPK and PI3K families. Yet, how changes to the global protein phosphoproteome contribute to the cellular response to IFN-I is unknown.

**Methods:**

The cerebral phosphoproteome of mice with brain-targeted chronic production of the IFN-I, IFN-α, was obtained. Changes in phosphorylation were analyzed by ontology and pathway analysis and kinase enrichment predictions. These were verified by phenotypic analysis, immunohistochemistry and immunoblots. In addition, primary murine microglia and astrocytes, the brain's primary IFN-I-responding cells, were acutely treated with IFN-α and the global phosphoproteome was similarly analyzed.

**Results:**

We identified widespread protein phosphorylation as a novel mechanism by which IFN-I mediate their effects. In our mouse model for IFN-I-induced neurodegeneration, protein phosphorylation, rather than the proteome, aligned with the clinical hallmarks and pathological outcome, including impaired development, motor dysfunction and seizures. In vitro experiments revealed extensive and rapid IFN-I-induced protein phosphorylation in microglia and astrocytes. Response to acute IFN-I stimulation was independent of gene expression and mediated by a small number of kinase families. The changes in the phosphoproteome affected a diverse range of cellular processes and functional analysis suggested that this response induced an immediate reactive state and prepared cells for subsequent transcriptional responses.

**Conclusions:**

Our studies reveal a hitherto unappreciated role for changes in the protein phosphorylation landscape in cellular responses to IFN-I and thus provide insights for novel diagnostic and therapeutic strategies for neurological diseases caused by IFN-I.

**Supplementary Information:**

The online version contains supplementary material available at 10.1186/s12974-021-02277-x.

## Background

Inflammation of the central nervous system (CNS) is a fundamental response to a wide range of stimuli ranging from virus infection to tumors and neurodegenerative diseases. A key component of the inflammatory response is the type I interferons (IFN-I), which have both protective and detrimental effects [1]. Importantly, increasing evidence points to a fundamental role for IFN-I in ageing and neurodegeneration [2–4]. In a recent study, Roy and colleagues (2020) demonstrated that IFN-I drive neuroinflammation in Alzheimer’s disease (AD) and mediate synapse loss [3]. At its extreme, IFN-I-mediated CNS inflammation and neurodegeneration are known as “*cerebral type I interferonopathies*” [5, 6]. They include genetic diseases (e.g., Aicardi–Goutières Syndrome, AGS), chronic and congenital infections (e.g., HIV, toxoplasma, and cytomegalovirus) and autoinflammatory disorders (e.g., neurological manifestations of systemic lupus erythematosus, SLE) [6–8]. Importantly, the mechanisms of how IFN-I drive neurological diseases are unclear, making causal treatment difficult.

The IFN-I are a large family of cytokines that includes multiple IFN-α subtypes and a single IFN-β [9]. The cellular effects of IFN-I are driven through the coordinated regulation of the expression of a large number of genes known as interferon regulated genes (IRGs). All IFN-I signal through the Janus kinase (JAK)/signal transducer and activator of transcription (STAT) pathway [10]. Canonical activation of the pathway leads to the phosphorylation of the transcription factors STAT1 and STAT2 that form a trimolecular complex with interferon regulatory factor 9 (IRF9). This complex, termed interferon stimulated gene factor 3, is transcriptionally active and is primarily responsible for mediating the expression of IRGs [10–12]. However, the canonical JAK/STAT pathway may only represent the tip of the iceberg when it comes to the cellular mechanisms by which IFN-I actions are mediated. The existence of alternative IFN-I-activated signaling pathways via mitogen-activated protein kinase (MAPK) and phosphatidylinositol 3-kinase [9, 13] suggests that we understand very little of the complexity of the IFN-I system and that IFN-I may use additional, as yet undefined mechanisms to mediate cellular responses.

Advancements in mass spectrometry-based techniques provide an excellent opportunity to uncover novel networks of protein interaction and regulation on a system-wide level. Using a global phosphoproteomic approach, we determined the global phosphoproteome in the CNS of transgenic mice with CNS-restricted production of IFN-α, termed GIFN39 mice [14, 15]. These mice recapitulate the key clinical and pathological changes seen in patients with cerebral type I interferonopathies [8]. We discovered that the changes in the phosphorylation landscape of the CNS in transgenic mice at early-stage disease aligned with disease features seen in mice with late-stage disease. The changes were largely driven by a small number of kinase families including MAPKs, cyclin-dependent kinases (CDKs), casein kinases (CKs) and calcium calmodulin kinases (CaMKs). Furthermore, the phosphoproteome of acutely IFN-α-treated microglia and astrocytes—the primary immune responding cells of the CNS—revealed an immediate reactive state that prepared these cells for the subsequent transcriptomic response. Functional comparison of the in vivo and in vitro phosphoproteomes suggested that microglia and astrocytes are prominent contributors to disease in cerebral type I interferonopathies. In summary, here we identify a novel mechanism for the regulation of the cellular response to IFN-I. Importantly, this mechanism is largely independent of altered transcription and may serve as a novel indicator for diagnostic and therapeutic purposes targeting particular kinases in a cell-type-specific manner.

## Methods

### Mice

Transgenic GIFN39 mice (originally obtained from the Scripps Research Institute, La Jolla, CA, USA, where they were developed by I. L. Campbell) are described previously [14, 15] and were bred and maintained in-house. Mice were maintained under specific-pathogen free conditions at the animal facility of the University of Sydney. For in vitro experiments, cells were isolated from cortices of 1–4-day old wildtype (WT) C57Bl/6 mice. For ex vivo experiments, 8- and 16-week-old mice were euthanized with isoflurane, measured, brains weighed and the cerebellum dissected and flash frozen. For histological analyses, brains were fixed in 10% neutral buffered formalin overnight and then paraffin-embedded.

### Primary microglia and astrocyte purification

Mixed glial cultures and subsequent purification into microglia and astrocytes were prepared as described previously [16]. Briefly, single-cell suspensions of papain digested cortices were plated on poly-D-lysine coated 75 cm^2^ flasks and incubated at 37 °C in a humidified incubator with 5% CO_2_ until confluent in media (Dulbecco's modified Eagle medium with high glucose, 10% fetal bovine serum, 100 U/ml penicillin and 10 μg/ml streptomycin). Microglia were collected by mechanical shaking. Astrocytes were purified by CD11b-negative selection following manufacturer’s instructions (130-049-601, Miltenyi Biotec, Macquarie Park, NSW, Australia). Flow cytometry with anti-CD11b (101211, Biolegend, San Diego, CA, USA) was used to determine astrocyte purity after sorting with an allowance of 0.5% CD11b^+^ contaminating cells. Purified microglia from three 75 cm^2^ flasks were pooled into one 75 cm^2^ flask and purified astrocytes were seeded at six million cells per 75 cm^2^ flask.

### IFN-α treatment of primary microglia and astrocytes

At 80–90% confluence, microglia and astrocytes were washed with media and incubated in media with or without 1,000 U/ml IFN-α_11_ (130-093-130, Miltenyi Biotec) at 37 °C in a humidified incubator with 5% CO_2_. Treatment was stopped with two washes of ice-cold PBS and protein extracted in lysis buffer.

### Phosphoproteomics

For both tissue and cells, phosphopeptides and non-phosphorylated peptides were obtained from the same animals or cell samples as outlined in Additional file 1: Fig. S1. For all samples, protein was extracted in urea lysis buffer (6 M urea (U6504, Sigma-Aldrich, Castle Hill, NSW, Australia), 2 M thiourea (88810, Sigma-Aldrich), 20 U/ml Benzonase (E1014, Sigma-Aldrich) with 1 × Protease Inhibitor Cocktail III (539,134, Merck, Bayswater, VIC, Australia) and 1 × Phosphatase Inhibitor Cocktail II (524,625, Merck) by hand-held homogeniser (Omni, Kennesaw GA). For microglia, protein from ten 75 cm^2^ flasks, and for astrocytes, protein from two 75 cm^2^ flasks, was pooled by methanol–chloroform precipitation and resuspended in urea lysis buffer. Cysteine residues were reduced in the presence of 1,4-dithiothreitol and subsequently alkylated with iodoacetamide. Following tryptic digestion, 250 µg of peptide was labelled with one of four isobaric tags (iTRAQ, Sciex, Mt Waverly, VIC, Australia; TMT, Thermo Fisher Scientific, Waltham, MA) as listed in Table S1. Phosphopeptides were enriched according to [17, 18], producing three peptide populations (singly and multiply phosphorylated and non-phosphorylated). Singly phosphorylated and non-phosphorylated peptides were further fractionated using offline hydrophilic interaction chromatography generating 10 fractions each. Identification and quantification were performed on an LTQ-Orbitrap (QE plus, ThermoFisher Scientific, North Ryde, NSW, Australia) mass spectrometer in data dependent mode. All experiments were performed in duplicate. Data were analyzed using Proteome Discoverer (Version 2.2, ThermoFisher Scientific) and searched using an in house MASCOT server (Version 2.4) against the UniProt Mus musculus database (database version July 2018) with the following parameters: maximum 2 missed cleavages, 20 ppm mass error (MS) and 0.2 Da mass error (MS/MS); iTRAQ was searched as a fixed modification; variable modifications included phosphorylation (Ser, Thr, Tyr), carbamidomethyl (Cys), oxidation (Met), acetylation (protein N-term) and cyclisation (Glu and Asp). False discovery rate of 0.01 was applied for non-phosphorylated peptides, which was relaxed to 0.05 for singly and multiply phosphorylated peptides, to allow for the labile nature of the modification. All experiments were performed in duplicate with moderate to strong correlation (Additional file 1: Fig. S2). Normalization of iTRAQ/TMT reporters was calculated using the sum of all intensities approach across all phosphorylated and non-phosphorylated peptide spectral matches (PSM) within a given experiment, prior to calculation of ratios using WT or 0 min IFN-α stimulated as denominator. Log_2_ ratios and population wide mean and standard deviation were calculated for each PSM using a total reporter ion intensity-dependent ranking for z-score calculation, adapted from [19]. The weighted average was used to compare median z-score and fold change across duplicate labelling experiments for each phosphosite and phosphopeptide (phosphoproteome), and protein (non-phosphorylated only). For analysis of proteome, significance was defined as a median z-score ≥ 1.96 or ≤ − 1.96 (non-phosphorylated, 95% confidence interval) and ≥ 1 or ≤ − 1 (68.3% confidence interval) for phosphopeptides and phosphosites. A minimum of five PSMs per protein was used a further confidence threshold for regulated non-phosphorylated proteins. Regulation was defined with a fold change ≥ 1.5 or ≤ − 1.5.

### Bioinformatics

Relative abundance of proteins were estimated by their NSAF as described in [20]. Ontology enrichment of biological processes (GOTERM_BP_DIRECT) and pathways (KEGG_PATHWAY) was performed with Database for Annotation, Visualization and Integrated Discovery (DAVID) v6.8 [21, 22]. Canonical pathways, upstream analysis and disease functions were analyzed by Ingenuity Pathway Analysis (IPA) (QIAGEN Inc., https://www.qiagenbioinformatics.com/products/ingenuity-pathway-analysis) [23]. Kinase prediction analyses utilized motif-X [24, 25] with changed parameters of foreground and background of the IPI mouse proteome and a motif length of 15; in vivo group-based prediction system (v1.0) [26] and PhosphoSitePlus, March 2017 [27]. The activity score of “activity, induced” or “activity, inhibited” for each kinase was calculated by calculating the sum of the multiplication of the log_2_ fold change of each significant PhosphoSitePlus-annotated phosphosites on the kinase with its PSM and + 1 if activity was induced or − 1 if activity was inhibited and then divided by the sum of the PSMs of those phosphosites. This score reflects the cumulative effect of the phosphosites on the kinase to an induced or inhibited state compared with the basal status weighted on fold change and normalized to abundance. A threshold score of 0.53 reflects a log_2_ of a 1.5-fold change in activity and if positive, an increase, and if negative, a reduction in activity. Proteins were considered as kinases as defined in [28]. Heatmaps, principle component analysis (PCA) and some plots were generated in R [29] using packages gplots [30], ggplot2 [31] and VennDiagram [32] and the princomp function. Summary of gene ontologies were extracted using REVIGO [33]. Sequences of mouse and human STATs were aligned with CLUSTAL O (1.2.4) [34] and annotated using information from PhosphoSitePlus with focus on transcription induction, inhibition, alteration and intracellular localization. IRGs were identified using the Interferome database [35].

### Histology

Paraffin Sections (5 μm) were deparaffinized and rehydrated in graded ethanol. Antigens were unmasked with 25 mM Tris pH 9 (for GFAP), 25 mM Tris pH 8 and 0.05% SDS (for Iba1) or 10 mM citrate pH 6 in 0.05% Tween-20 (for CD3, neurofilament and cleaved caspase-3) in a vegetable steamer for 45 min. Sections were incubated in 0.03% H_2_O_2_ for 10 min and blocked in 1% goat serum with 0.1% Triton-X and 0.05% Tween-20 in PBS for 20 min. Primary antibodies against GFAP (Z0334, Agilent, Santa Clara, CA, USA; 1:1,000 dilution), Iba1 (019–19,741, Wako Pure Chemical Industries, Osaka, Japan; 1:1,000 dilution), CD3 (ab16669, Abcam, Melbourne, VIC, Australia; 1:200 dilution), cleaved-caspase-3 (9661, Cell Signaling Technologies; 1:300 dilution) and neurofilament (N0142, Sigma-Aldrich; 1:200 dilution) were incubated overnight at 4 °C. Following washing, sections were incubated with biotinylated secondary anti-rabbit and anti-mouse antibodies (BA-1000 and BA-2000, Vector Laboratories, Burlingame, CA, USA) followed by VECTASTAIN Elite ABC HRP Kit (PK-7200, Vector Laboratories). Sections were developed with 3,3′-diaminobenzidine with or without Ni enhancement (SK-4100, Vector Laboratories), prepared for dual stains or counterstained with Mayer’s Hematoxylin and mounted. For alizarin red S (ARS) post-immunostaining, sections were incubated with 2% ARS pH 4.2, destained in acetone, washed in 1:1 acetone:xylene and in xylene and then a cover slip was applied. Sections were view with the DM4000B microscope (Leica Macquarie Park, NSW, Australia) and imaged with Spot Imaging Software (Spot Imaging, Sterling Heights, MI, USA). For Iba1-ARS visualization of microglia and calcification, images were acquired at various focus depths and blended in Adobe Photoshop. Brightness and contrast were globally adjusted using Adobe Photoshop.

### Immunoblotting

Total protein for immunoblotting was isolated from the cerebellum of mice or treated microglia and astrocytes in 50 mM Tris pH 7.5, 150 mM NaCl, 1 mM EDTA, 1% sodium deoxycholate, 1% Triton-X 100, 0.1% SDS and 1 × Protease Inhibitor Cocktail III and 1 × Phosphatase Inhibitor Cocktail II. Proteins were separated on a 10% Tris–glycine acrylamide gel, transferred onto polyvinylidene fluoride membranes, blocked with 5% skim milk or 5% bovine serum albumin in 137 mM NaCl, 20 mM Tris–HCl (pH 7.4) and 0.1%(v/v) Tween-20 and probed using primary and secondary antibodies. Primary antibodies used were pY701-STAT1 (7649; 1:2,000 dilution), pS727-STAT1 (9177; 1:2,000 dilution), STAT1 (9172; 1:2,000 dilution), STAT2 (4597; 1:1,000 dilution), pY705-STAT3 (9131; 1:2,000 dilution), STAT3 (4904; 1:2,000 dilution), pY694-STAT5 (9351; 1:250 dilution), pT202/204-MAPK1/3 (9101; 1:2,000 dilution), pT180/pY182-MAPK14 (9211; 1:1,000 dilution), MAPK14 (9212; 1:1,000 dilution), pT183/pY185-MAPK8/9 (9255; 1:1,000 dilution), MAPK8/9 (9252; 1:1,000 dilution), pS473-AKT (4058; 1:1,000 dilution), AKT (4691; 1:2,000 dilution) (all from Cell Signaling Technologies, Danvers, MA, USA), pY689-STAT2 (07–224; 1:1,000 dilution, Merck Millipore), GAPDH (MAB374, Merck Millipore; 1:30,000 dilution), MAPK1/3 (M5670, Sigma-Aldrich; 1:40,000 dilution) and STAT5 (sc835, Santa Cruz Biotechnology, Dallas, Texas, USA; 1:200 dilution). Secondary antibodies used were horseradish peroxidase conjugated anti-rabbit (sc2004, Santa Cruz; 1:30,000 dilution) and peroxidase conjugated anti-mouse (A0168, Sigma-Aldrich; 1:10,000 dilution). Chemiluminescence (WBKLS0500, Merck Millipore) was detected in the ChemiDoc XRS + (BioRad Laboratories, Gladesville, NSW, Australia) and relative proteins levels were quantified by densitometry using Fiji [36]. To reduce and account for variations across membranes, membranes were incubated together with the same solutions and imaged at the same time, while identical samples were included on all membranes and normalized to each other. Source data available in (Additional file 2).

### Experimental design and statistical analysis

Both genders of mice were analyzed when available in the colony and there was no previously observed effect of gender in these mice with outcome of disease [14, 15]. Only female mice were used for phosphoproteomic analyses to reduce chance of variability with mixed genders. Samples sizes were based on previous studies and are listed in figure legends. Statistical analyses of different data sets are outlined in figure legends and were performed in GraphPad Prism, version 8 (La Jolla, CA, USA). Briefly, comparisons of survival curves were made by log-rank test and comparisons between genotypes and age were made by two-way ANOVA with Tukey’s post-test. Normality of residuals and homogeneity of variance were based on QQ plots and homoscedasticity plots, respectively. A *P* ≤ 0.05 was considered significant.

## Results

### Chronic IFN-α signaling in vivo upregulates proteins that drive an inflammatory response

**Fig. 1:**
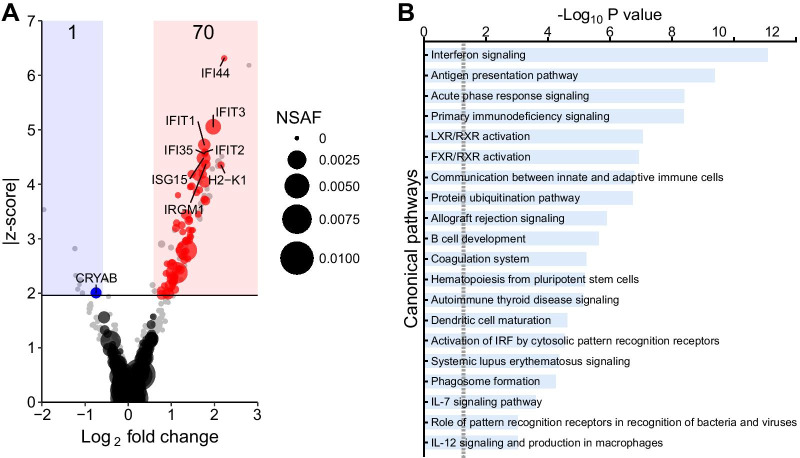
Regulated protein in the cerebellum of GIFN39 mice are characteristic of an interferon-induced immune response. **A** Distribution of protein abundances (nonphosphopeptides; blue: reduced and red: increased; number of significantly altered proteins is indicated at the top) in GIFN39 *vs* WT cerebella and their relative abundance estimated by NSAF (grey: protein abundance below threshold). Data of cerebella from WT (*n* = 4) and GIFN39 (*n* = 4) mice were analyzed from two runs with *n* = 2 per genotype in each. **B** Top 20 pathways associated with the regulated proteins in the cerebellum of GIFN39 mice compared with WT mice, determined by IPA. The dashed line: *P* = 0.05

To determine global changes in protein levels and changes in protein phosphorylation in the CNS of GIFN39 vs WT mice, we isolated proteins from the cerebella and performed mass spectrometry on nonenriched (proteome) and enriched phosphopeptides (phosphoproteome) as described in material and methods. The cerebellum was chosen as it has the highest level of transgene expression in the CNS [14]. Eight-week-old GIFN39 mice were used as they show only mild inflammatory changes and minimal signs of neurodegeneration, focusing our analysis on primarily IFN-α-driven responses rather than the compounded effects from leukocyte infiltration and neurodegeneration. A total of 5,616 proteins were detected with one protein less abundant and 70 proteins more abundant (|z-score|≥ 1.96 and |fold change|≥ 1.5) in the proteome of the cerebellum of GIFN39 mice compared with WT mice (Fig. 1A and Additional file 1: Tables S1 and S2). The protein with reduced abundance was α-crystallin B chain (CRYAB) and is a non-classical chaperone preventing protein aggregation in stress responses [37]. Yet, its role in neurodegenerative and neuroinflammatory diseases is undetermined. Of the 71 proteins, 82% are regulated by IFN-I, extrapolated using the Interferome database. The other proteins are antibody subunits and endopeptidase inhibitors. Using IPA, enriched canonical pathways consisting of the proteins with elevated abundance in the cerebellum of GIFN39 mice were associated with interferon signaling, antigen presentation, acute phase response and interestingly, but less significant, SLE signaling (Fig. 1B).

### Extensive protein phosphorylation in GIFN39 mice gives deeper insights into disease processes

**Fig. 2:**
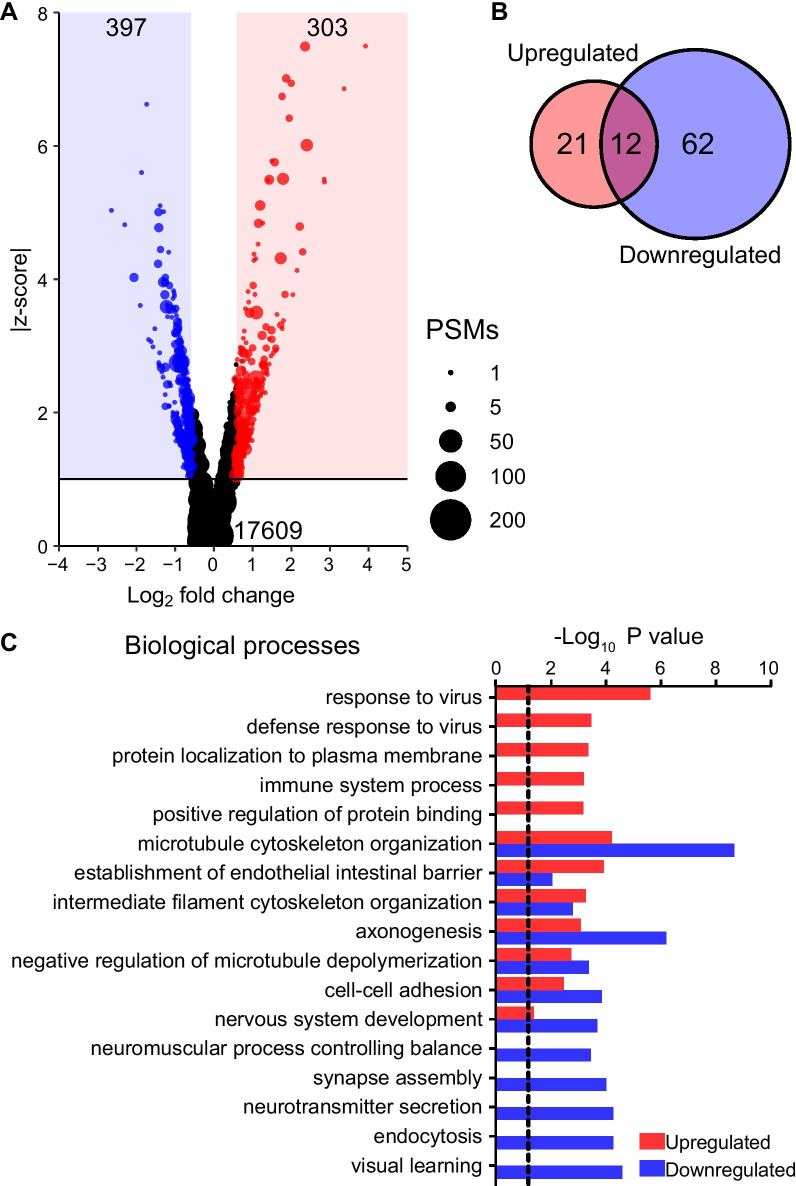
Phosphoproteome in the cerebellum of GIFN39 mice is highly regulated. **A** Several hundred sites had increased (red) or reduced (blue) phosphorylation in the cerebellum of GIFN39 mice compared with WT mice. Total number of detected phosphosites (bottom) and significantly regulated phosphosites (top) are indicated. Phosphosite abundance indicated by PSMs. **B** Comparison between significant biological processes, determined by DAVID, associated with increased or decreased phosphorylation with **C** the top processes shown. Dotted line: *P* = 0.05

Analysis of the phosphoproteome of cerebella from WT and GIFN39 mice revealed 17,609 phosphosites on 4,748 proteins and a strong correlation between replicates (Additional file 1: Fig. S2 and Table S1). Comparing the phosphoproteome from GIFN39 with WT mice, 303 phosphosites on 203 proteins showed significantly increased phosphorylation and 397 phosphosites on 295 proteins decreased phosphorylation (Fig. 2A). Of these regulated phosphoproteins, 22 proteins were significantly increased in abundance, while one had reduced abundance. To dissect the contribution of increased and/or decreased phosphorylation in biological processes, functional analysis was performed on each regulated set of proteins and compared using the DAVID. A large number of processes were associated with decreased phosphorylation (Fig. 2B) reflecting the greater number of dephosphorylated phosphosites. Proteins with increased phosphorylation were associated with responses to virus and immune processes, whereas proteins with reduced phosphorylation were largely associated with neuron-related functions and endocytosis (Fig. 2C). Top processes with increased and decreased phosphorylation included regulation of the cytoskeleton and cell–cell interactions, such as the endothelial barrier. Thus, while the ontologies of the regulated proteome were associated primarily with immune responses, the regulated phosphoproteome reflected both immune responses and additional non-immune functions. Furthermore, the functional analysis of the phosphoproteome provided a greater insight into the regulated processes than the proteome.

### The CNS-phosphoproteome of GIFN39 mice reflects their phenotype and disease outcome

**Fig. 3:**
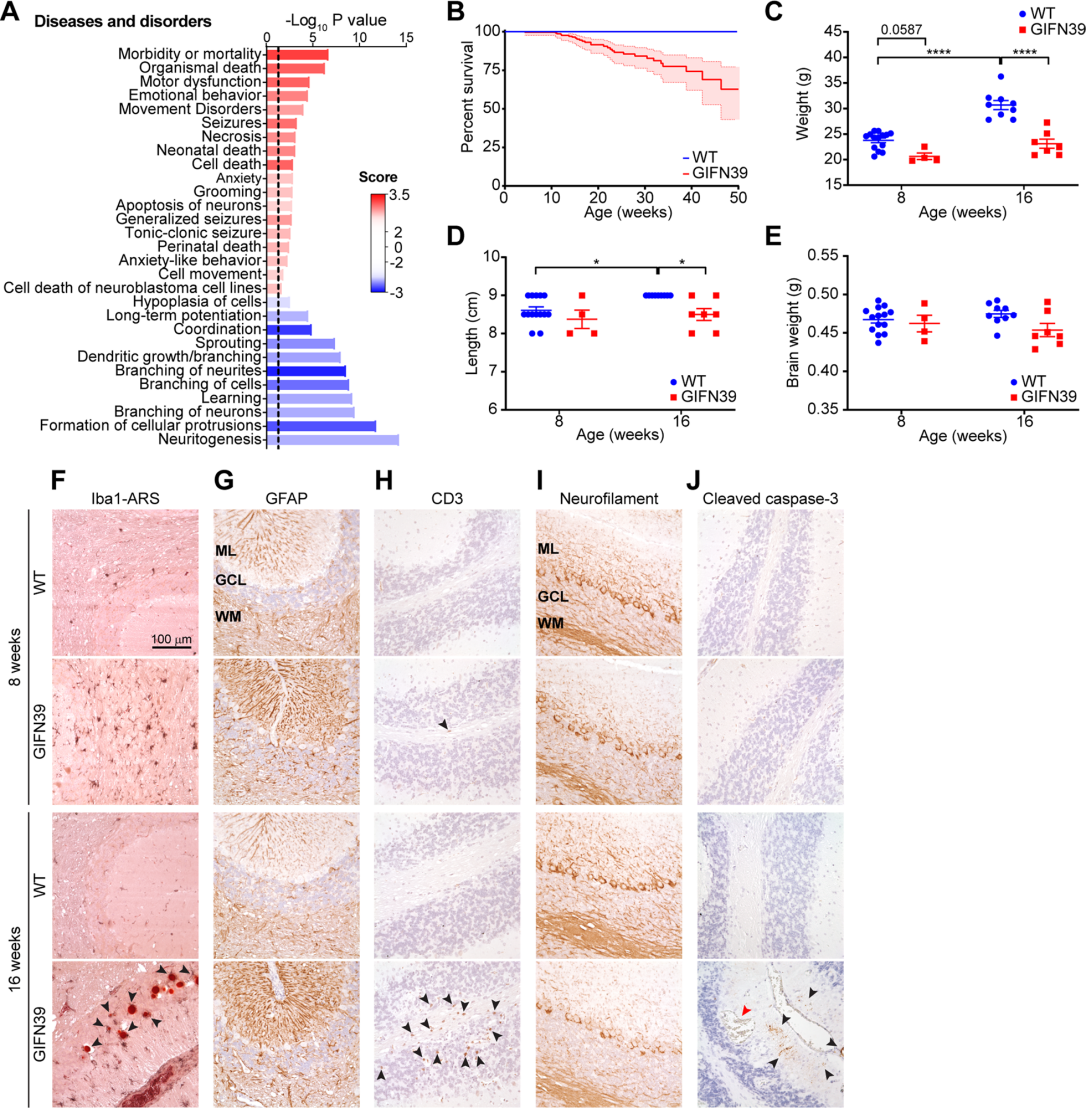
Predicted diseases from the CNS-phosphoproteome of GIFN39 mice and associated clinical and pathology outcomes. **A** Activation state of diseases and disordered enriched from the regulated phosphoproteome of GIFN39 mice. Positive score (red) indicates increased activity and negative score (blue) indicates decreased activity of the clinical and pathological signs of disease. **B** Progressive reduction in survival of GIFN39 mice (total *n* = 317) compared to WT littermates (total *n* = 376), *P* < 0.001 by log-rank test. Data presented as mean ± 95% confidence interval (shaded). **C** Body weight, **D** length of mice from nose to tail base and **E** wet brain weight was reduced in male GIFN39 mice compared with WT mice and with age (*n* = 4**–**13 per genotype per age). Data presented with mean ± SEM. * P < 0.05, ** P < 0.01 and **** P < 0.0001 between indicated samples as determined by two-way ANOVA with Tukey’s post-test. n.s.: not significant. Neuropathology was investigated in the cerebellum of 8 and > 16-week-old GIFN39 mice and WT littermates. (F) Dual Iba1 (brown/black) and Alizarin Red S (ARS; red; indicated by arrowheads) stain for microglia and calcium deposits. (G) GFAP stain for astrocytes. (H) CD3 stain for T cells, indicated by arrowheads. (I) Neurofilament stain to reveal neurons. (J) Cleaved caspase-3 stain for apoptotic cells indicated by black arrowheads. Red arrowhead indicates an aneurysm. Representative immunohistochemical and histological stains of cerebella of WT and GIFN39 of 8-week-old and 16-week-old mice (*n* = 4 mice per genotype per age). ML: molecular layer, GCL: granule cell layer and WM: white matter

Next, we further investigated the regulated global phosphoproteome in GIFN39 mice using IPA. By grouping phosphoproteins into protein–protein networks, IPA determined the enrichment of diseases and disorders associated with the networks and calculated an activation score. The significantly activated diseases and disorders included mortality, motor dysfunction, emotional behavior, seizures, cell death and anxiety-like behavior (Fig. 3A). By contrast, processes associated with neuronal function, such as long-term potentiation and neurite branching, were significantly reduced.

To validate these enriched diseases and disorders of the 8-week-old GIFN39 mouse, we investigated their clinical and pathological outcomes at eight and 16 weeks of age. GIFN39 mice had a significantly reduced survival compared with WT littermates (Fig. 3B) and were smaller in weight and length at both ages while remaining the same weight across 8 weeks (Fig. 3C, D). In addition, the average brain weight of GIFN39 mice was less at 16 weeks of age compared with WT mice (Fig. 3E), suggesting brain atrophy. Furthermore, at 16 weeks of age, GIFN39 mice had moderate to severe ataxia, displayed wild running and jumping and occasional convulsive seizures, as previously described [14, 15].

Pathologically, GIFN39 mice developed perivascular calcification, where some microglia were observed wrapping the deposits (Fig. 3F). Microglia were also observed to be highly ramified with increased Iba1 staining compared with WT mice, at both ages. There was increased GFAP immunoreactivity in the cerebellum of GIFN39 mice (Fig. 3G), which was also reflected in the proteomic data (Additional file 1: Table S2) and by immunoblot (Additional file 1: Fig. S3). A progressive increase in CD3^+^ T cell infiltrates was observed with age in the GIFN39 mice, while T cells were not observed in the cerebellum of WT mice (Fig. 3H). There was reduced neurofilament staining of Purkinje cells and dendrites in the molecular layer in 16-week-old GIFN39 mice compared with WT mice indicating neurodegeneration (Fig. 3I). In addition, there was staining for cleaved caspase-3 only observed in 16-week-old GIFN39 mice (Fig. 3J) revealing increased apoptosis. Large vascular lumens were observed in the brains of GIFN39 mice at 16 weeks of age indicating the presence of aneurysms (Fig. 3J). Together, the clinical and pathological observations in aged GIFN39 mice validate those identified by the phosphoproteome at early-stage disease.

We next probed for the phosphorylation status of various STATs as a proxy to changes in the global phosphoproteome with age. Canonical IFN-I signaling relies on Y701-STAT1 phosphorylation, which was significantly elevated compared with the cerebellum of WT mice at both ages (Additional file 1: Fig. S3). In addition, pS727-STAT1, which is considered to modulate STAT1 activity [38, 39], and total STAT1 were significantly increased in abundance and ratio in GIFN39 mice compared with WT mice. There was no detectable increase in STAT2 activation (pY689-STAT2) but total STAT2 was increased in GIFN39 mice compared with WT mice. In addition to STAT1 and STAT2, STAT3 and STAT5 have also been implicated to contribute to IFN-I signaling [9, 13]. Accordingly, STAT3 was activated (pY705-STAT3) in the cerebellum of GIFN39 mice and increased with age. However, activation of STAT5 was not detected. Thus, these findings show chronic IFN-I signaling in the brains of GIFN39 mice and further activation of alternative signaling pathways with age, possibly associated with neurodegeneration.

### Extensive protein phosphorylation in GIFN39 mice is associated with only a few kinase families

**Fig. 4:**
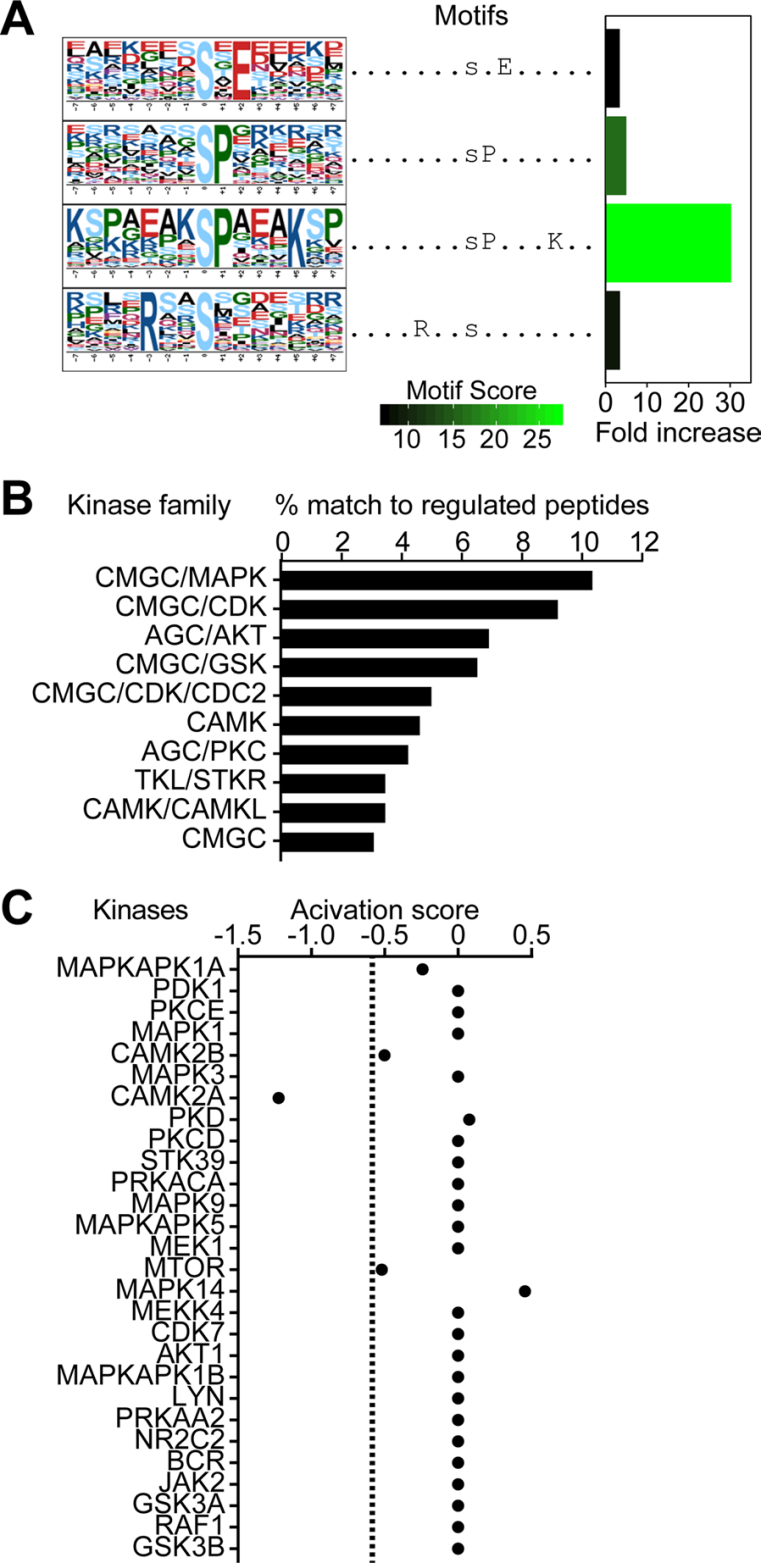
Few kinase families regulate the phosphoproteome in GIFN39 mice. **A** Sequence logos and fold increase of enriched motifs of the upregulated phosphopeptides calculated by motif-X. Kinase family and matched consensus substrate motif: MAPK/CDK (sP and sP…K), CK (s.E) and CaMK (R..s). **B** Top 10 kinase families predicted based on substrate motif matches and protein**–**protein interaction. Percentages of the upregulated phosphopeptides associated with a kinase compared with the total number of phosphopeptides associated with a kinase family are shown. **C** Kinase activity score of kinases calculated from the abundance, fold change (GIFN39 *vs* WT) and role, extracted from PhosphoSitePlus, of each phosphosite on the kinase. Dashed line: threshold score equivalent to a 1.5-fold reduced activity

The results suggest changes in the phosphoproteome is involved the progression of the disease. We next investigated which kinases drive the phosphorylation of proteins using three in silico approaches. There are no established strategies for identifying phosphatases, which mediate protein dephosphorylation, largely due to dissimilarities of substrate-motifs of targets [40]. The first approach to identify kinases was substrate-centric, whereby enriched peptide motifs with increased phosphorylation were extracted with motif-X. Enriched motifs were matched to consensus kinase-substrate motifs to identify active kinase families. Four serine-centric motifs were enriched and associated with the MAPK/CDK (sP and sP…K), CK (s.E) and CaMK (R..s) families (Fig. 4A). The MAPK and CDK families share a common proline-directed phosphorylation site [41], resulting in both families matching to similar motifs. The second approach predicted kinase families based on their association to substrate motifs and the protein–protein interactome. The number of substrates linked to each kinase family was normalized to the number of phosphorylated peptides associated with a kinase family to give the percentage of phosphopeptides targeted by a kinase family. Similar to the first approach, the MAPK family was predicted to phosphorylate the largest percentage of phosphosites in the cerebellum of GIFN39 mice, followed by CDK and AKT (Fig. 4B). In addition, 27% of phosphosites were not matched to a kinase. The third approach was kinase-centric, calculating a kinase activation score. This score estimates the overall activity of a kinase from the cumulative effect of each phosphosite in relation to its abundance and effect on the kinase—whether it increased or inhibited activity, extracted from PhosphoSitePlus. Interestingly, there was a slight increase in MAPK14 activity and a slight decrease in activity from CaMK2A, CaMK2B and mTOR (Fig. 4C). However, a large number of phosphosites did not have annotations resulting in only 28 of all 250 kinases identified in our data being assigned an activation score. Although there are limitations to each approach including variable kinase-substrate specificities, multiple kinases targeting the same protein and most phosphosites having undefined roles [42], the three approaches identified the same few core kinase families, with the MAPK family having the most prominent presence in the regulation of the cerebellar phosphoproteome in GIFN39 mice. In support of this, immunoblots for MAPK1/3 and MAPK14 showed phosphorylation at their canonical activation sites in the cerebellum of GIFN39 mice, while phosphorylation of MAPK8/9 was not detected (Additional file 1: Fig. S3). The phosphosites for MAPK1/3 and MAPK14 were also detected in the phosphoproteome indicating basal activation. Furthermore, there was slight increased MAPK3 activation in GIFN39 mice compared with WT mice.

### The proteome of primary microglia and astrocytes aligns with their expected biological roles

To determine, the contribution of specific cell types to the phosphoproteome changes in the CNS of GIFN39 mice, we next analyzed the proteome and phosphoproteome of microglia and astrocytes, which are dominant IFN-I responding CNS-resident cells [16, 43]. The phosphoproteome is sensitive to signaling with rapid global changes occurring within minutes [44–48]. This excluded the isolation and ex vivo analysis of cell types from the brain of mice. Hence, we investigated the response to IFN-α in vitro in primary murine microglia and astrocytes. We treated microglia or astrocytes with IFN-α for 0, 5, 15 and 30 min to determine their direct cellular response to IFN-α. The short treatment ensured a largely transcription-independent response in the absence of secondary signaling from IRGs or secreted cytokines and non-physiological responses attributed to a cultured system.

Proteomic post-hoc analysis showed high purity of our microglia and astrocyte cultures, based on the relative protein abundance of cell-type-specific markers (Additional file 1: Fig. S4A, B). Comparing the proteomes (Table S1), 3,017 proteins were common between microglia and astrocytes, while 743 and 1,240 proteins were specific to microglia and astrocytes, respectively. The biological processes and pathways associated with the common proteins mapped to fundamental cellular processes including gene transcription, translation and metabolism (Table S3). Microglia-specific proteins mapped to their known roles as primary resident immune cells: immune-related processes and inflammation. For astrocytes, the top processes and pathways included cell–cell contact, cell size and brain development. Furthermore, top ten transcriptional regulators common to both cell types were associated with homeostasis, metabolism and proliferation (Table S4). The top ten microglia-associated transcriptional regulators were involved with myeloid lineage, immune function and homeostasis, whereas astrocyte-associated transcriptional regulators were linked to cell migration and proliferation, and homeostasis. Thus, common and cell-type-specific transcriptional regulators align well with the top biological processes and pathways of the total proteome further reflecting the cell-type-specific roles of these cells in the CNS.

### IFN-α induces extensive and cell-type-specific changes of the phosphoproteome in microglia and astrocytes

**Fig. 5:**
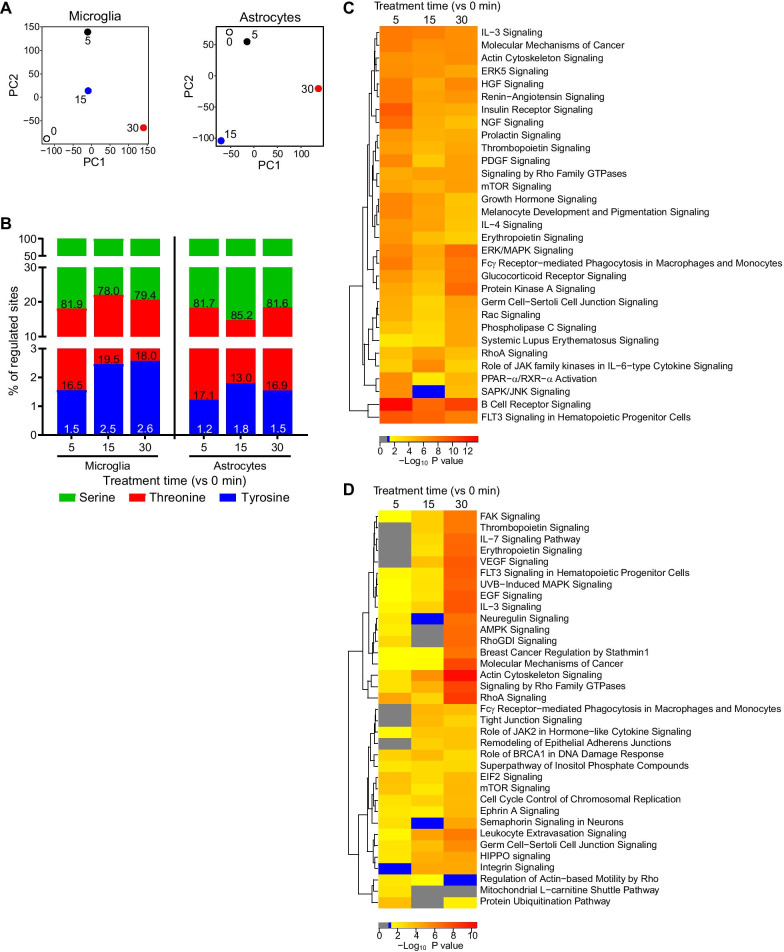
IFN-α extensively modulates phosphorylation profiles of microglia and astrocytes in a cell-type-specific manner. **A** PCA of untreated and IFN-α-treated microglia and astrocytes indicate distinct phosphorylation changes with treatment times (indicated by the number adjacent to each point). **B** Percentage of regulated phospho-serine, -threonine and -tyrosine residues in treated microglia and astrocytes compared with untreated cells. Numbers indicate percentages. Hierarchically clustered heatmap of the top 15 significant canonical pathways at each timepoint (*vs* 0 min) in **C** microglia and **D** astrocytes determined by IPA. Grey: non-enriched, blue: non-significantly enriched and yellow to red bars are significantly enriched pathways

Investigating the IFN-α response in microglia and astrocytes by PCA of the fold changes of all phosphosites revealed distinct changes in the phosphoproteome across treatment duration and between cell types (Fig. 5A). In IFN-α-treated microglia, the overall change to the phosphoproteome was greater than in astrocytes (Additional file 1: Fig. S5). In microglia, after 5 min of IFN-α treatment, 1,480 sites on 817 proteins had significantly increased phosphorylation levels and 1,091 sites on 726 proteins had reduced phosphorylation levels, when compared with untreated cells (|z-score|≥ 1 and |fold change|≥ 1.5). Numbers decreased after 15 min before increasing again after 30 min treatment. By contrast, in astrocytes, 109 sites on 99 proteins had increased phosphorylation and 348 sites on 295 proteins had decreased phosphorylation levels after 5 min treatment. The number increased further after 15 and 30 min IFN-α treatment. In addition, although tyrosine phosphorylation is key in mediating signaling of IFN-I, only 1.2–2.6% of regulated residues were tyrosine, with greater regulation in microglia compared with astrocytes (Fig. 5B). The other two residues, serine and threonine, were most abundantly regulated. This reflects the abundance of each of these residues in the proteome determined in previous large-scale phosphoproteomic studies [42, 46]. This demonstrated that microglia and astrocytes responded rapidly and extensively to IFN-α and that the response in microglia was more wide-ranging than in astrocytes.

Next, we analyzed the functional correlates associated with the IFN-α-regulated phosphoproteome using IPA. The top 15 significant pathways in microglia were similar across all treatment timepoints and were associated with immunity, actin dynamics, proliferation, survival and apoptosis and various intracellular signal transduction and metabolism pathways (Fig. 5C). In contrast to microglia, the astrocyte response showed greater variability over time and included cell membrane interactions and remodeling, translation, proliferation, survival and apoptosis, and actin dynamics (Fig. 5D).

### Microglia and astrocytes show distinct IFN-α-induced phosphorylation dynamics of the JAK/STAT pathway

**Fig. 6:**
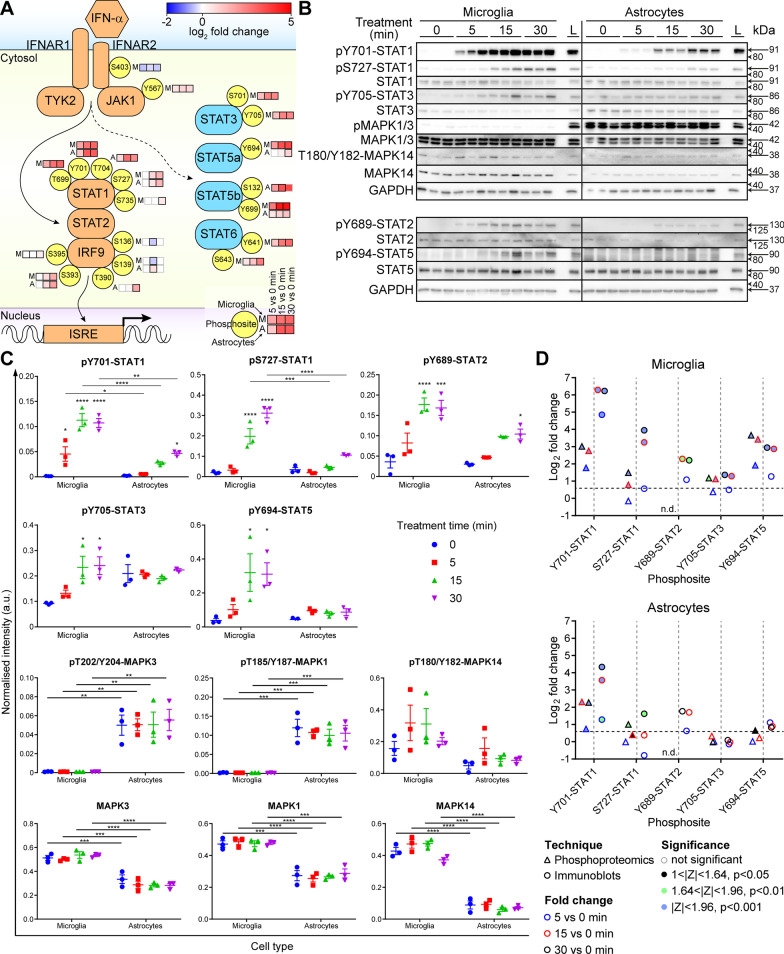
IFN-α induces cell type-specific phosphorylation of JAK/STAT pathway components in treated microglia and astrocytes. **A** IFN-α-associated JAK/STAT signaling pathway depicting phosphosites and their log_2_ fold change at 5, 15 and 30 min compared with 0 min. Phosphosites were included if they were significantly regulated at one timepoint. Grey boxes: did not reach significance. **B** Immunoblot of whole protein lysates from IFN-α-treated microglia and astrocytes (*n* = 3 per cell type per timepoint). Sample “L” was a cross-membrane loading control of a pooled protein from all 30 min IFN-α-treated microglia and astrocyte samples. **C** Densitometric quantifications of immunoblots. Mean ± SEM are shown. * P < 0.05, ** P < 0.01, *** P < 0.001 and **** P < 0.0001 compared to the respective 0 min of the cell type or between indicated samples as determined by two-way ANOVA with Tukey’s post-test. **D** Similar trends in fold change between phosphoproteomics and immunoblots. Fold changes calculated from immunoblots are based on the mean intensities at 0 min of treatment and significance determined by two-way ANOVA with Tukey’s post-test. Significance set at |z-score|≥ 1 for phosphoproteomics and *P* < 0.05 for immunoblots. Dashed horizontal line indicates log_2_ of a 1.5-fold change. n.d.: not detected

To further investigate the differential signaling kinetics in microglia and astrocytes, we focused on the JAK/STAT pathway. Due to the nature of mass spectrometry, not all phosphosites can be identified owing to detection limits of peptide length, peptide physico-chemical properties, peptide abundance and use of data-dependent acquisition [49–51]. Nevertheless, a comprehensive and unbiased survey of the global phosphoproteome is achievable. Expectedly, pY701-STAT1, an activation marker of IFN-I signaling, was markedly increased in both cell types following IFN-α treatment (Fig. 6). pS727-STAT1 levels similarly increased with treatment time but with delayed kinetics and a lower magnitude aligning with its proposed role of modulating the activity of STAT1 [38, 39]. Together, IFN-α treatment induced the expected increase of the canonical markers of IFN-I activation with time [52–54] revealing the two-phase regulation of the microglia global phosphoproteome was not an artifact.

Interestingly, additional phosphosites (T699, T704 and S735) were regulated on STAT1 by IFN-α (Figs. 6A and Additional file 1: Fig. S6). These sites lie in the transactivation domain suggesting regulation of transcriptional activity of STAT1 and that pT704 may be involved in dimerization of STATs [55]. Extrapolation of the function of these sites from annotated STATs indicate a role for modulating activity and protein-complex formation (Additional file 1: Fig. S7A). Following Y701 phosphorylation, STAT1 dimerizes with tyrosine-phosphorylated STAT2 and IRF9 to regulate gene expression. While no phosphosites were detected by mass spectrometry on STAT2, likely owing to above mentioned reasons, phosphorylation of the canonical Y689 was identified by immunoblot (Fig. 6B–D). By contrast, multiple sites were phosphorylated on IRF9, detected in the GIFN39 cerebellum and IFN-α-treated cells (Fig. 6A, S6 and S7B), which may modulate transcription though protein-complex formation as the residues lie in the C-terminal IRF-associated domain (Additional file 1: Fig. S7B). In addition to STAT1 and STAT2, phosphoproteomic analysis identified activation of STAT3, STAT5a, STAT5b and STAT6 in microglia and STAT5a and STAT5b in astrocytes in response to IFN-α (Fig. 6A). Of these, tyrosine phosphorylation of pY705-STAT3 and pY689-STAT5 was confirmed by immunoblot (Fig. 6B–D). Together, these findings reveal dichotomous signaling kinetics between microglia and astrocytes in the acute response to IFN-α. Furthermore, the data suggest novel ways in which canonical IFN-I signaling is modulated between these cell types.

### Diversity of IFN-α-regulated phosphosites is modulated by a limited number of kinases

**Fig. 7:**
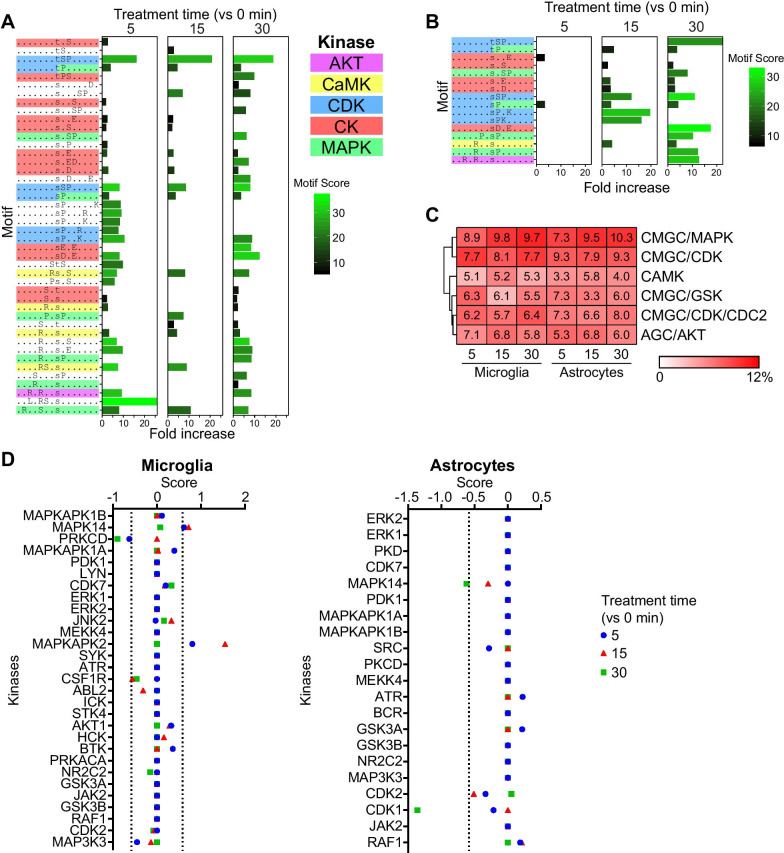
Only few predicted kinase families drive IFN-α-induced phosphorylation in both microglia and astrocytes. Fold enrichment of motifs extracted from phosphopeptides with increased phosphorylation using motif-X in **A** microglia and **B** astrocytes. Kinase families were predicted by matching the enriched motif with their consensus substrate motif and are color coded. **C** Top kinase families from treated microglia and astrocytes based on substrate motif matches and protein**–**protein interaction. Percentages of the upregulated phosphopeptides associated with a kinase compared with the total number of phosphopeptides associated with a kinase family per treatment and cell type are shown. **D** IFN-α-regulated activity score of kinases calculated from the abundance, fold change and role, extracted from PhosphoSitePlus, of each phosphosite on the kinase. Dashed line: threshold score equivalent to a 1.5-fold change in activity

We next investigated the contributing kinases to the increased phosphorylation measured by IFN-α treatment. To identify kinases associated with the IFN-α-induced changes in phosphorylation, we used the same three in silico approaches as above. In microglia, the enriched motifs matched to substrates of MAPK/CDK, CK, CaMK and AKT (Fig. 7A). One third of enriched motifs had no clear match to a kinase. While fewer enriched motifs were identified in the treated astrocytes, the same five kinase families were matched (Fig. 7B), indicating similar core IFN-I signaling pathways. In support, the second approach similarly identified MAPK and CDK families as the key kinases contributing to IFN-α-induced phosphorylation in microglia and astrocytes (Fig. 7C). Other top predicted kinase families were CaMK, glycogen synthase kinase (GSK) and AKT. The third approach identified MAPK14 and its downstream target MAPKAPK2 to have 1.5-fold increased activity in IFN-α-treated microglia (Fig. 7D). By contrast, IFN-α treatment in astrocytes reduced activity of MAPK14. However, activation of members of the MAPK family was detected by immunoblots in both cell types following IFN-α treatment resulting in a slight but non-significant increase in MAPK14 phosphorylation (Additional file 1: Fig. S8). Moreover, there was a significantly higher abundance of these kinases in microglia compared with astrocytes (Additional file 1: Fig. S8). In addition, activity of PRKCD and CDK1 was reduced after 30 min of IFN-α treatment in microglia and astrocytes, respectively. Together, the MAPK family was a major contributor to IFN-α-induced protein phosphorylation in both microglia and astrocytes. This supports the notion seen in vivo of a core IFN-α response being largely driven by these kinases, while differences in phosphorylation kinetics and cellular response between the two cell types reflects the changes in activity of each kinase.

### Astrocytes rather than microglia play a diverse role in the regulated global phosphoproteome in GIFN39 mice

**Fig. 8:**
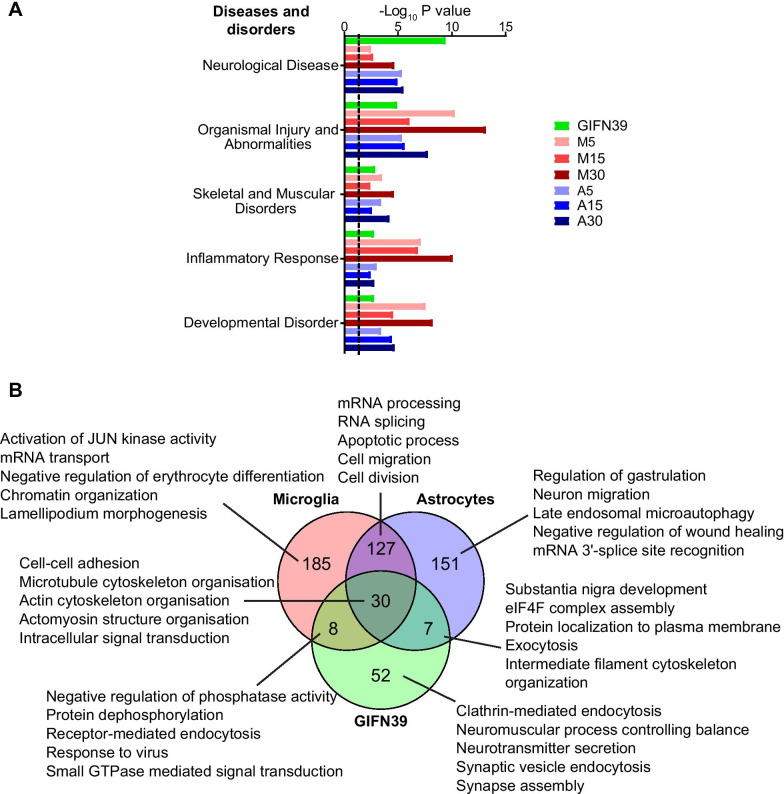
Contribution of acute IFN-α signaling to the regulated cerebellar phosphoproteome in GIFN39 mice. **A** Top five enriched diseases and disorders categories determined by IPA between the regulated phosphosites of the GIFN39 cerebellum and IFN-α-treated microglia (“M”) and astrocytes (“A”). Treatment times indicated next to the cell type. Dotted lines: *P* = 0.05. **B** Comparison of the biological processes from GIFN39 cerebellum and IFN-α-treated microglia and astrocytes as determined by DAVID. Shown are total number of significant biological processes with the top five biological processes of each segment being listed

We next extrapolated the contribution of direct IFN-α signaling in microglia and astrocytes in the disease to GIFN39 mice. Of note, the presence of neurons and other cells in vivo likely reduces detection of microglia- and astrocyte-specific proteomes in the cerebellum. In addition, other cytokines and mediators of inflammation in the inflamed brains of GIFN39 mice would also modulate the detected phosphoproteome. Furthermore, to investigate the contribution of IFN-α signaling by cell type in vivo would be difficult as the phosphoproteome would rapidly change during cell isolation. Hence, we restricted our comparison to functional ontologies. Although, acutely-treated cells will likely differ in their response compared with microglia and astrocytes in the brains of GIFN39 mice, comparison of the diseases and disorders in treated microglia and astrocytes suggested that IFN-α signaling in microglia and astrocytes contributed to the top diseases and disorders enriched in the regulated cerebellar phosphoproteome of GIFN39 mice (Fig. 8A). Delineating the functional contribution, the enriched biological processes of each of the regulated phosphoproteomes were compared. Overall, 46% of processes enriched in the phosphoproteome of GIFN39 mice were also enriched in IFN-α-treated cells (Fig. 8B). To summarize ontology terms, semantics were used to combine related terms [33]: in common, was nervous system development including establishment of the endothelial intestinal barrier, cytoskeleton organization, protein autophosphorylation, endocytosis, positive regulation of protein binding, intracellular signal transduction, cell–cell adhesion and negative regulation of neuron apoptotic processes. Processes contributed by microglia include response to virus, positive regulation of microtubule polymerization, receptor mediated endocytosis and protein dephosphorylation. Processes contributed by astrocytes include eukaryotic translation initiation factor 4F complex assembly, exocytosis, substantia nigra development and endoplasmic reticulum calcium ion homeostasis. Expectedly, neuron-associated processes were exclusive to the cerebella phosphoproteome of GIFN39 mice. Together, these results indicate the cell-type-specific contribution from the direct IFN-α signaling in astrocyte, rather than microglia, have a more diverse contribution to the changes in the cerebellar phosphoproteome in GIFN39 mice.

## Discussion

Type I interferons have both beneficial and detrimental actions in the CNS ranging from protection against infections to mediating inflammation and neurodegeneration. Although the wide spectrum of actions of IFN-I are known to involve IRGs, here we identify a novel mechanism by which IFN-I regulate the cellular response that is independent of transcription. We demonstrate that in addition to regulating gene expression, IFN-I induce widespread changes in protein phosphorylation in vivo. These changes preceded severe disease in a mouse model for cerebral type I interferonopathies and—importantly—reflected the cardinal features of the clinical and pathological disease. Furthermore, microglia and astrocytes are key cellular components of this phosphoproteome response. Finally, in silico analysis revealed that a limited number of kinase families mediated a large proportion of the protein phosphorylation, identifying them as potential targets for future therapeutic intervention of IFN-I-mediated neuroinflammatory diseases.

Chronic production of IFN-α in young GIFN39 mice resulted in altered phosphorylation of nearly 500 proteins despite the absence of pronounced disease or overt neurodegeneration. Strikingly, the phosphoproteome accurately reflected the development of pathological features and disease outcome in GIFN39 mice. Functional analysis revealed enriched disease categories in the phosphoproteome of GIFN39 mice that are also present in humans with cerebral type I interferonopathies [56–58], suggesting that widespread protein phosphorylation also contributes to these diseases. Accordingly, the total number of proteins showing differential phosphorylation in GIFN39 *vs* WT mice was comparable to findings in mouse models of AD and several human degenerative and inflammatory diseases including AD and SLE [59–61]. While increased phosphorylation in the GIFN39 cerebellum was broadly associated with a response to virus infection, reduced phosphorylation was linked to neuronal functions. A similar observation has been made in AD [59] and various other neurodegenerative diseases [62, 63] suggesting that dysregulated protein phosphorylation plays a significant role in a wide range of neurodegenerative and neuroinflammatory diseases.

In contrast to the widespread changes in protein phosphorylation, only 71 proteins showed altered abundance in cerebella from 8-week-old GIFN39 mice compared with WT mice. This low number is probably due to a combination of factors, including the mice not yet showing prominent disease, and the relative high proportion of neurons in the cerebellum [64] which have a very limited response to IFN-I [43]. Of the 71 proteins identified in our study several are associated with vascular aneurysms [65], such as fibronectin, which are key pathological features observed in GIFN39 mice, patients with cerebral type I interferonopathies and during IFN-I treatment [57, 66]. In addition, antigen presentation and autoimmunity were prominently featured, corroborating studies that have identified autoantibodies in type I interferonopathies [67, 68]. Of note, similar to blood mononuclear cells from patients with SLE [61, 69], a systemic type I interferonopathy, the diversity of biological processes was limited in the total proteome compared with the phosphoproteome, demonstrating the importance of posttranslational modifications for regulating cellular responses. Nevertheless, changes in the proteome—like those of the phosphoproteome—were highly specific and correlated well with key pathological features of GIFN39 mice and patients with type I interferonopathies, alike.

Microglia and astrocytes are highly responsive to IFN-I, regulating a significantly greater number of IRGs when compared with neurons [16, 43]. Here we found that acute treatment of microglia and astrocytes in vitro resulted in extensive changes in protein phosphorylation. In addition to a common IFN-I response, both cell types showed cell-type-specific responses that were reflective of their biological roles in vivo. In microglia, immunity-associated intracellular signaling was enriched, paralleling recent transcriptomic findings [16] and further highlighting their role as the principal immune cell of the CNS. Interestingly, SLE signaling was only enriched in microglia, suggesting a role for microglia in the CNS manifestations of this disease. Accordingly, microglia are a major source of IFN-I in CNS-lupus [70]. By contrast, top enriched pathways in IFN-α-treated astrocytes underscored the role of these cells in blood–brain barrier regulation and integrity, and contribution to leukocyte extravasation. Accordingly, loss of IFN-I signaling in astrocytes increases blood brain barrier permeability during infection with a neurotropic virus [71]. Together, this suggests that microglia and astrocytes are prominent contributors to the global response seen in the CNS in vivo.

The changes in protein phosphorylation in microglia and astrocytes occurred within 5 to 30 min and were thus largely independent of de novo gene expression. Furthermore, several signaling pathways enriched in the acute phosphoproteome were also identified in the transcriptome of these cells in response to 12 h treatment with IFN-α [16], suggesting that the acute global phosphorylation-dependent response to IFN-α guides the transcriptomic response. Thus, as phosphorylation preceded transcription and translation upon IFN-α stimulation, changes in protein phosphorylation likely prime microglia and astrocytes and instigate rapid host protection responses in a transcription-independent manner.

To address the molecular basis underlying the increased protein phosphorylation we performed in silico analysis of kinase motifs and activities. Unexpectedly, these studies revealed that only a limited set of kinases belonging to the MAPK/CDK, CK, CaMK and AKT families likely mediated the majority of the observed phosphorylation events in vivo and in vitro. These kinase families have previously been associated with the cellular response to IFN-α [53, 72]. It is tempting to speculate that differential activities and abundances of members of the MAPK family may contribute to the lower expression of several IRGs in astrocytes compared with microglia in response to IFN-α [16]. Of note, while mass spectroscopy and immunoblots revealed no obvious differences in phosphorylation of these kinases apart from MAPK3 and MAPK14, it is conceivable that additional post translational modifications, such as acetylation of MAPK14 [73], also contribute to increased kinase activity. A role for the MAPK pathway in regulating cellular effects to IFN-α is further supported by the presence of S727 phosphorylation of STAT1 in GIFN39 mice and IFN-α-treated cells, which is mediated by MAPK14 [74–76]. It will be interesting to determine if different IFN-I subtypes activate the same kinases. While all IFN-I share the same components of the canonical signalling pathway, they bind to the receptor with different kinetics and affinities [77, 78]. It is conceivable that this could result in differences in downstream kinase activation and IFN-I-subtype specific responses [79]. Our studies also identified a number of novel phosphosites in the canonical IFN-I signaling molecules STAT1 and IRF9. In addition to the critical Y701-STAT1, three phosphosites on STAT1 previously not reported to be IFN-α-regulated, as well as the known site S727, were seen in IFN-α-treated cells. Interestingly, T699, T704 and S735 phosphorylation was cell-type specific, suggesting that these sites may contribute to the distinct responses in microglia and astrocytes. Since these residues lie within the transactivation domain of STAT1 it is conceivable that they regulate transcriptional activity either directly or through recruitment of co-activators. Accordingly, crystal structures indicate that T704-STAT1 could be involved in STAT1 dimerization [55]. Similarly, IRF9 was phosphorylated at multiple sites. Unlike other IRF family members, IRF9 is constitutively active [80] and does not require phosphorylation of the C-terminal region. However, based on sequence similarities the identified phosphosites on IRF9 could indicate the presence of a so far unappreciated signaling network that modulates interferon stimulated gene factor 3 activity.

Importantly, our results further suggest a clear dichotomy in IFN-I signaling, where the changes in the cellular phosphoproteome are mediated mostly by members of the MAPK/CDK, CK, CaMK and AKT kinase families, whereas transcriptomic changes are largely dependent on canonical JAK/STAT signaling. These findings open up new opportunities for the development of kinase-specific drugs as a mean to modulate IFN-I-mediated neuroinflammation and neurodegeneration. Accordingly, in recent years, several kinase inhibitors have been approved for the treatment of a range of degenerative diseases, immune disorders and cancers [81, 82]. For example, JAK inhibitors (e.g., baricitinib, tofacitinib) have been used to treat patients with type I interferonopathies [83], while LRRK2 and CDK5 inhibitors are being explored as treatments for various neurodegenerative disorders [81]. While targeting the MAPK pathway has been less successful due to toxicity [84], identifying specific members, such as MAPK14, above may allow for the development of more specific inhibitors with less side effects.

## Conclusions

This study provides evidence for a novel mechanism for IFN-I action that involves so far unappreciated and extensive changes in the protein phosphorylation landscape affecting a diverse range of cellular processes. The changes in the phosphoproteome not only preceded disease in a mouse model for cerebral type I interferonopathy but aligned with the clinical and pathological outcome. In microglia and astrocytes, IFN-α-induced protein phosphorylation was rapid and occurred in a cell-type-specific manner highlighting the biological roles of both cell types in the CNS and their potential contribution to IFN-I-induced disease. In addition to preparing the cells for the subsequent transcriptomic response, the changes in the phosphoproteome induced a reactive state in the cells. The changes are mediated, in parallel to the canonical IFN-I JAK/STAT signaling pathway, by a limited number of kinase families. These studies reveal a hitherto unappreciated role for global protein phosphorylation in response to IFN-I and provide novel insight for the development of diagnostic and therapeutic strategies for diseases of the CNS and periphery caused by IFN-I.

## Supplementary Information

**Additional file 1.** Additional tables and figures.

**Additional file 2.** Source data.

## Data Availability

The mass spectrometry proteomics data have been deposited to Zenodo (http://doi.org/10.5281/zenodo.4422055 and http://doi.org/10.5281/zenodo.4433003) and to the ProteomeXchange Consortium via the Proteomics Identification (PRIDE) [85] partner repository with the data set identifier PXD014443 and http://doi.org/10.6019/PXD014443. Total quantified proteins, phosphopeptides and phosphosites of WT vs GIFN39 cerebella and IFN-α-treated microglia and astrocytes (Table S1) has been deposited to Zenodo (https://doi.org/10.5281/zenodo.4287788).

## Abbreviations

AD: Alzheimer’s disease
AGS: Aicardi-Goutières syndrome
ANOVA: Analysis of variance
ARS: Alizarin Red S
CaMK: Calcium calmodulin kinase
CDK: Cyclin-dependent kinase
CK: Casein kinase
CNS: Central nervous system
CRYAB: α-Crystallin B chain
DAVID: Database for Annotation, Visualization and Integrated Discovery
GAPDH: Glyceraldehyde 3-phosphate dehydrogenase
GFAP: Glial fibrillary acidic protein
GIFN39: GFAP-IFNα mice
GSK: Glycogen synthase kinase
Iba1: Ionized calcium-binding adapter molecule 1
IFN-α: Interferon-alpha
IFN-I: Type I interferons
IPA: Ingenuity Pathway Analysis
IRF: Interferon regulator factor
IRGs: Interferon regulated genes
iTRAQ: Isobaric tags for relative and absolute quantitation
JAK: Janus kinase
KEGG: Kyoto Encyclopedia of Genes and Genomes
MAPK: Mitogen-activated protein kinase
MAPKAPK2: MAPK-activated protein kinase 2
n.d.: Not detected
NSAF: Normalized spectral abundance factor
PCA: Principle component analysis
PRKCD: Protein kinase C delta type
pS: Phospho-serine
PSM: Peptide spectral match
pT: Phospho-threonine
pY: Phospho-tyrosine
SLE: Systemic lupus erythematosus
STAT: Signal transducer and activator of transcription
WT: Wildtype
