# Supplementary material for: A novel phosphoproteomic landscape evoked in response to type I interferon in the brain and in glial cells
Source: J Neuroinflammation. 2021 Oct 16;18:237. doi: 10.1186/s12974-021-02277-x (PMC8520650; doi:10.1186/s12974-021-02277-x)
Supplement: Supplementary file 1 — Additional file 1. Additional tables and figures. [file 12974_2021_2277_MOESM1_ESM.docx]

**A novel phosphoproteomic landscape evoked in response to type I interferon in the brain and in glial cells**

Barney Viengkhou^1^, Melanie Y White^2^, Stuart J Cordwell^2^, Iain L Campbell^1^, Markus J Hofer^1^*

**List of additional materials:**

Table S1. Total quantified proteins, phosphopeptides and phosphosites of WT vs GIFN39 cerebella and IFN-α-treated microglia and astrocytes. Available at www.zenodo.org (https://doi.org/10.5281/zenodo.4287788).

Table S2. Regulated proteins in the cerebellum of GIFN39 mice compared with WT mice.

Table S3. Top ten significant biological processes and KEGG pathways of proteins common and unique between microglia and astrocytes using DAVID.

Table S4. Top predicted transcription factors of microglia and astrocytes identified by IPA.

Figure S1. Flow diagram for generating non-phosphoproteomic and phosphoproteomic datasets.

Figure S2. Correlation of fold changes of phosphosites between duplicate and replicate runs.

Figure S3. Immunoblot for STATs and their activation in the cerebellum of GIFN39 mice.

Figure S4. Cell purity of microglia and astrocyte primary cultures.

Figure S5. Significantly regulated phosphosites after IFN-α-treatment in microglia and astrocytes.

Figure S6. Summed intensities of detected phosphosites on STAT1 and IRF9.

Figure S7. Annotated sequence alignment of STAT and IRF proteins with detected IFN-α-regulated phosphosites.

Figure S8. Immunoblots for changes in activation of a subset of MAPK members in IFN-α-treated microglia and astrocytes.

Table S2. Regulated proteins in the cerebellum of GIFN39 mice compared with WT mice.

| **Protein accession** | **Gene** | **Log_2_ GIFN39/WT** | **z-score** | **NSAF** |
| --- | --- | --- | --- | --- |
| Q8BV66 | *Ifi44* | 2.23 | 6.31 | 2.99E-05 |
| P01901 | *H2-K1* | 2.15 | 4.35 | 7.53E-05 |
| Q64345 | *Ifit3* | 1.97 | 5.06 | 1.11E-03 |
| Q60766 | *Irgm1* | 1.80 | 4.36 | 2.28E-04 |
| P01837 | *Igkc* | 1.79 | 3.70 | 1.89E-04 |
| Q64112 | *Ifit2* | 1.78 | 4.59 | 1.02E-04 |
| Q9D8C4 | *Ifi35* | 1.77 | 4.56 | 5.30E-05 |
| P01887 | *B2m* | 1.77 | 4.04 | 4.24E-04 |
| P32261 | *Serpinc1* | 1.77 | 3.72 | 1.03E-04 |
| Q64282 | *Ifit1* | 1.76 | 4.73 | 5.51E-04 |
| Q64339 | *Isg15* | 1.74 | 4.48 | 7.53E-04 |
| Q61107 | *Gbp4* | 1.73 | 4.18 | 1.38E-04 |
| Q6Q899 | *Ddx58* | 1.72 | 4.29 | 1.39E-04 |
| Q9QZ85 | *Iigp1* | 1.65 | 3.89 | 7.34E-05 |
| Q00897 | *Serpina1d* | 1.62 | 3.45 | 4.89E-05 |
| P42225 | *Stat1* | 1.62 | 4.17 | 4.55E-04 |
| P28063 | *Psmb8* | 1.58 | 3.85 | 8.24E-05 |
| Q61703 | *Itih2* | 1.49 | 3.16 | 4.00E-05 |
| Q8R2Q8 | *Bst2* | 1.47 | 3.96 | 1.03E-04 |
| O08573 | *Lgals9* | 1.47 | 4.19 | 1.29E-04 |
| P01029 | *C4b* | 1.46 | 3.16 | 3.34E-05 |
| Q62293 | *Tgtp1* | 1.44 | 3.04 | 4.87E-05 |
| Q07797 | *Lgals3bp* | 1.44 | 3.95 | 5.69E-05 |
| Q61702 | *Itih1* | 1.43 | 3.06 | 3.90E-05 |
| O35309 | *Nmi* | 1.41 | 3.35 | 4.83E-05 |
| P01897 | *H2-L* | 1.41 | 2.97 | 9.07E-05 |
| P01900 | *H2-d1* | 1.40 | 3.34 | 1.52E-04 |
| Q9R233 | *Tapbp* | 1.40 | 3.17 | 3.80E-05 |
| P24452 | *Capg* | 1.38 | 2.63 | 5.74E-05 |
| P03995 | *Gfap* | 1.35 | 2.79 | 2.37E-03 |
| P23953 | *Ces1c* | 1.35 | 2.97 | 1.41E-04 |
| Q3U5Q7 | *Cmpk2* | 1.34 | 3.44 | 3.05E-04 |
| P01865 | *IGH-1a* | 1.34 | 2.77 | 6.35E-05 |
| P01902 | *H2-K1* | 1.33 | 2.96 | 1.24E-04 |
| P01869 | *Ighg1* | 1.30 | 2.77 | 3.21E-05 |
| P01867 | *Igh-3* | 1.30 | 2.71 | 3.75E-05 |
| Q9Z0E6 | *Gbp2* | 1.30 | 2.61 | 9.86E-05 |
| Q9Z2F2 | *Oasl2* | 1.26 | 3.13 | 3.98E-05 |
| Q61147 | *Cp* | 1.26 | 2.68 | 6.66E-05 |
| P98086 | *C1qa* | 1.24 | 3.25 | 9.28E-05 |
| E9Q555 | *Rnf213* | 1.21 | 2.88 | 1.62E-05 |
| P22599 | *Serpina1b* | 1.20 | 2.43 | 1.90E-04 |
| Q61704 | *Itih3* | 1.17 | 2.46 | 5.68E-05 |
| Q3UIR3 | *Dtx3l* | 1.17 | 2.79 | 4.39E-05 |
| Q03963 | *Eif2ak2* | 1.16 | 3.80 | 2.94E-05 |
| P07724 | *Alb* | 1.13 | 2.38 | 2.54E-03 |
| P01899 | *H2-D1* | 1.12 | 3.29 | 6.98E-05 |
| P11928 | *Oas1a* | 1.11 | 3.20 | 1.03E-04 |
| P01898 | *H2-Q10* | 1.10 | 2.29 | 3.89E-05 |
| P28665 | *Mug1* | 1.09 | 2.34 | 3.76E-05 |
| Q9DBD0 | *Ica* | 1.09 | 2.40 | 2.53E-05 |
| P16110 | *Lgals3* | 1.08 | 2.07 | 1.63E-04 |
| P11276 | *Fn1* | 1.08 | 2.24 | 1.43E-05 |
| P29788 | *Vtn* | 1.07 | 2.20 | 3.17E-05 |
| P51910 | *Apod* | 1.05 | 2.30 | 1.07E-04 |
| P50543 | *S100a11* | 1.03 | 2.26 | 4.38E-04 |
| O08677 | *Kng1* | 1.02 | 2.20 | 1.11E-04 |
| O35955 | *Psmb10* | 1.02 | 2.57 | 4.63E-05 |
| P97371 | *Psme1* | 1.02 | 2.53 | 3.35E-04 |
| P21958 | *Tap1* | 1.01 | 2.20 | 3.14E-05 |
| P36371 | *Tap2* | 1.00 | 2.34 | 3.60E-05 |
| P07758 | *Serpina1a* | 0.98 | 2.15 | 4.10E-04 |
| P20918 | *Plg* | 0.97 | 2.08 | 3.73E-05 |
| P28184 | *Mt3* | 0.93 | 1.99 | 4.46E-04 |
| Q62191 | *Trim21* | 0.90 | 2.54 | 3.22E-05 |
| Q91X72 | *Hpx* | 0.88 | 2.00 | 1.76E-04 |
| P01872 | *Ighm* | 0.88 | 2.37 | 4.45E-05 |
| Q3UPF5 | *Zc3hav1* | 0.84 | 2.14 | 5.07E-05 |
| P02802 | *Mt1* | 0.78 | 1.97 | 2.48E-04 |
| P97372 | *Psme2* | 0.77 | 2.05 | 1.48E-04 |
| P23927 | *Cryab* | -0.75 | -2.01 | 3.90E-04 |

Table S3. Top ten significant biological processes and KEGG pathways of proteins common and unique between microglia and astrocytes using DAVID.

|  | **Biological processes** | **Adjusted  p-value** |  | **KEGG pathways** | **Adjusted  p-value** |
| --- | --- | --- | --- | --- | --- |
| **Common** | Protein transport | 2.8E-68 |  | Spliceosome | 7.5E-37 |
|  | Cell-cell adhesion | 2.0E-56 |  | Biosynthesis of antibiotics | 7.3E-26 |
|  | mRNA processing | 1.4E-51 |  | Ribosome | 2.8E-25 |
|  | RNA splicing | 5.5E-48 |  | Carbon metabolism | 3.5E-20 |
|  | Translation | 1.5E-40 |  | RNA transport | 1.7E-17 |
|  | Transport | 3.6E-33 |  | Proteasome | 1.0E-16 |
|  | Vesicle-mediated transport | 9.3E-32 |  | Endocytosis | 5.7E-16 |
|  | Intracellular protein transport | 5.6E-29 |  | Metabolic pathways | 5.5E-14 |
|  | Protein folding | 4.5E-25 |  | Huntington's disease | 1.8E-13 |
|  | Metabolic process | 9.9E-22 |  | Protein processing in endoplasmic reticulum | 2.8E-12 |
| **Microglia** | Immune system process | 1.2E-10 |  | Osteoclast differentiation | 2.6E-12 |
|  | Innate immune response | 5.7E-08 |  | B cell receptor signaling pathway | 8.3E-08 |
|  | Positive regulation of phagocytosis | 1.3E-05 |  | F_c_ γ R-mediated phagocytosis | 1.9E-07 |
|  | Inflammatory response | 1.7E-04 |  | F_c_ ε RI signaling pathway | 2.0E-07 |
|  | Integrin-mediated signaling pathway | 1.7E-04 |  | Natural killer cell mediated cytotoxicity | 5.6E-06 |
|  | Phagocytosis | 1.6E-04 |  | Leishmaniasis | 2.0E-05 |
|  | Response to bacterium | 1.6E-03 |  | Staphylococcus aureus infection | 2.8E-04 |
|  | Leukocyte cell-cell adhesion | 6.8E-03 |  | Platelet activation | 4.6E-04 |
|  | Protein phosphorylation | 8.8E-03 |  | Phagosome | 6.1E-04 |
|  | B cell receptor signaling pathway | 8.8E-03 |  | Influenza A | 1.4E-03 |
| **Astrocytes** | Cell adhesion | 2.3E-09 |  | Focal adhesion | 1.8E-04 |
|  | Single organismal cell-cell adhesion | 4.6E-04 |  | Adherens junction | 4.3E-04 |
|  | Cilium morphogenesis | 1.3E-03 |  | Hippo signaling pathway | 1.6E-03 |
|  | Oxidation-reduction process | 3.1E-03 |  | Bacterial invasion of epithelial cells | 1.0E-02 |
|  | Brain development | 5.2E-03 |  | ErbB signaling pathway | 8.7E-03 |
|  | Cilium assembly | 1.3E-02 |  | Tight junction | 1.4E-02 |
|  | Regulation of rho protein signal transduction | 1.2E-02 |  | Metabolic pathways | 1.3E-02 |
|  | Actin cytoskeleton organization | 1.5E-02 |  | Wnt signaling pathway | 1.2E-02 |
|  | Lipid metabolic process | 2.3E-09 |  | Proteoglycans in cancer | 2.6E-02 |
|  | Cell migration | 4.6E-04 |  | Glycine, serine and threonine metabolism | 2.6E-02 |

Table S4. Top predicted transcription factors of microglia and astrocytes identified by IPA.

| **Common** | |  | **Microglia** | |  | **Astrocytes** | |
| --- | --- | --- | --- | --- | --- | --- | --- |
| **Transcription factor** | **p-value** |  | **Transcription factor** | **p-value** |  | **Transcription factor** | **p-value** |
| TP53 | 2.42E-34 |  | SPI1 | 6.54E-06 |  | YAP1 | 9.04E-06 |
| NFE2L2 | 9.74E-20 |  | FOXO4 | 1.91E-05 |  | TP53 | 7.64E-05 |
| KDM5A | 1.14E-14 |  | TRIM24 | 4.27E-04 |  | REL | 1.85E-03 |
| RB1 | 1.06E-12 |  | IRF7 | 1.45E-03 |  | VAX2 | 4.43E-03 |
| ARNT | 1.01E-09 |  | FOXO1 | 2.20E-03 |  | PAX6 | 8.68E-03 |
| RRP1B | 2.95E-09 |  | STAT6 | 2.36E-03 |  | KDM5A | 9.31E-03 |
| ESRRA | 3.55E-09 |  | CBFA2T3 | 3.04E-03 |  | ATF2 | 1.09E-02 |
| PML | 1.88E-08 |  | ZFPM1 | 3.10E-03 |  | ERG | 1.27E-02 |
| TFAM | 4.40E-08 |  | FOXO3 | 3.61E-03 |  | CLOCK | 1.65E-02 |
| NRF1 | 2.29E-07 |  | MED14 | 4.63E-03 |  | TCF7L2 | 1.72E-02 |


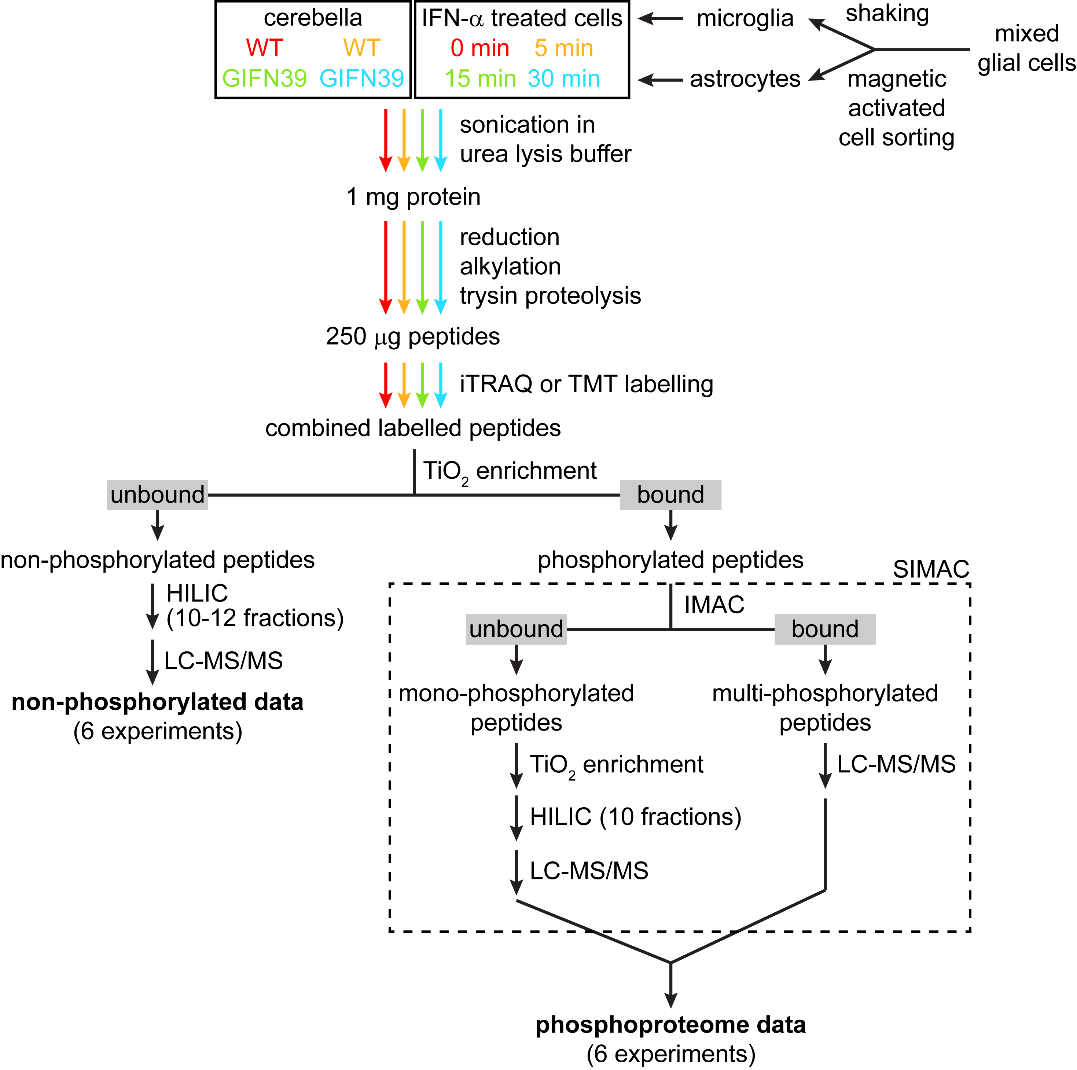


**Figure S1. Flow diagram for generating non-phosphoproteomic and phosphoproteomic datasets.** Samples (cerebella from WT or GIFN39 mice or IFN-α-treated microglia or astrocytes) were labelled and processed to obtain non-phosphorylated and phosphorylated peptides for mass spectrometry analysis as described in the materials and methods. A total of 6 experiments were performed with two sets of samples of the cerebella and IFN-α-treated microglia and astrocytes to generate data on the proteome and phosphoproteome.


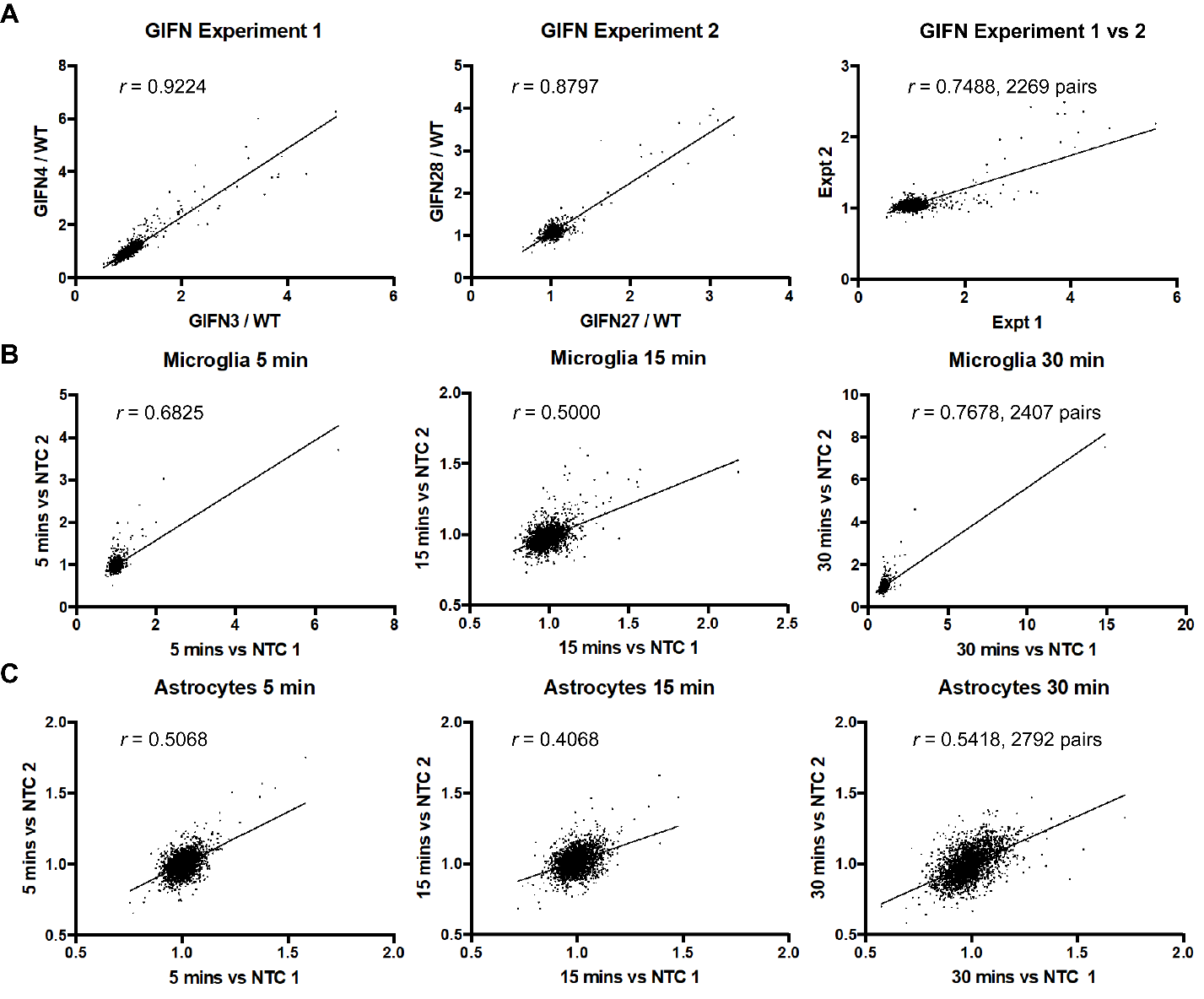


**Figure S2. Correlation of fold changes of phosphosites between duplicate and replicate runs.** Plots and Pearson’s correlation between replicates runs for (A) GIFN39 vs WT cerebella and duplicate runs of (B) microglia and (C) astrocyte treated with IFN-α at 5, 15 and 30 min vs 0 min. The number of proteins that were quantified across experiments are indicated as number of pairs in the right-hand column.


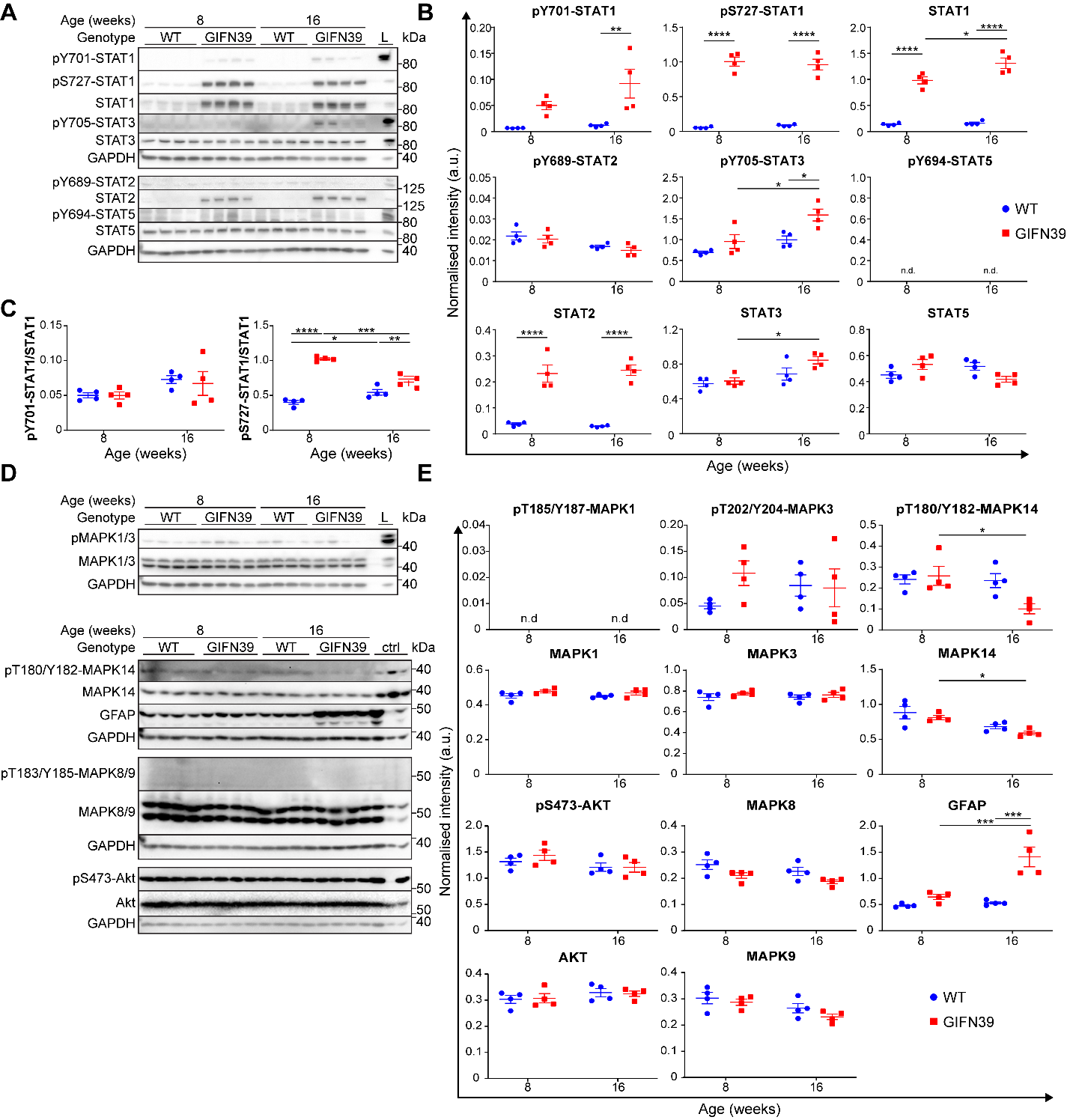


**Figure S3. Immunoblot for activation of STATs and kinases in the cerebellum of GIFN39 mice.** Immunoblots of cerebella protein lysates from WT and GIFN39 mice at 8 and 16 weeks of age (n = 4 per genotype per age) for activation of (A) STATs and (D) MAPK and level of total GFAP (GAPDH of top panel is equivalent to the top panel in (A)). Sample “L” was protein IFN-α-treated microglia and astrocyte. Controls (ctrl) in (D) is protein lysate, in order, from interleukin-6 overexpression in the cerebellum and IFN-α-treated microglia and astrocytes. (B, E) Densitometric quantification of immunoblots. Normalized to GAPDH. (C) Ratio of phosphorylated STAT1 to total STAT1. n.d.: not detected. Mean ± SEM are shown. *P < 0.05, **P < 0.01, ***P < 0.001 and ****P < 0.0001 between indicated samples as determined by two-way ANOVA with Tukey’s post-test.


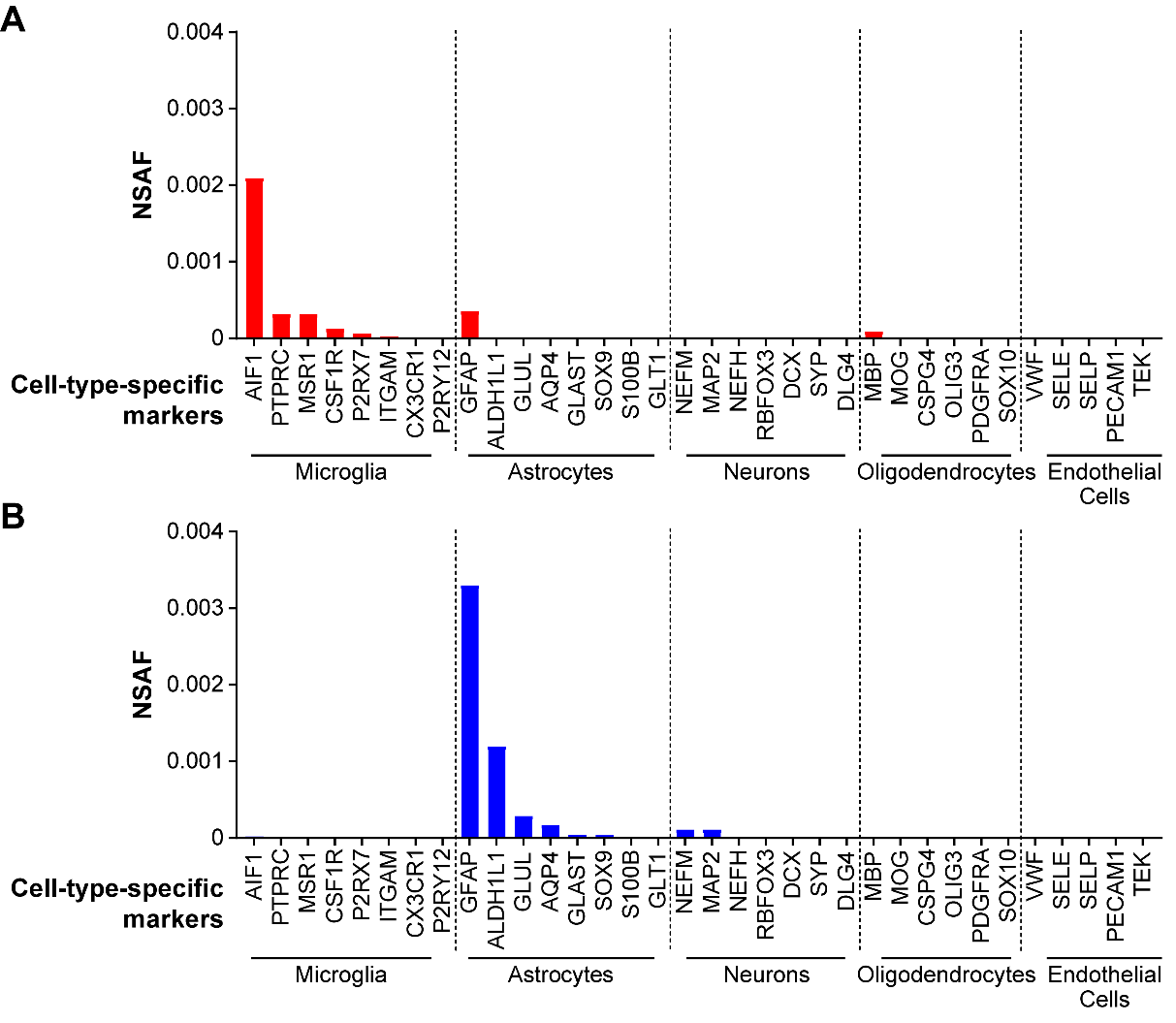


**Figure S4. Cell purity of microglia and astrocyte primary cultures.** Post-hoc cell purity analysis on the non-phosphorylated peptides (total proteome) revealed (A) microglia and (B) astrocyte cultures were of high purity based on their normalized spectral abundance factors (NSAF) estimates of known cell-specific markers.

**
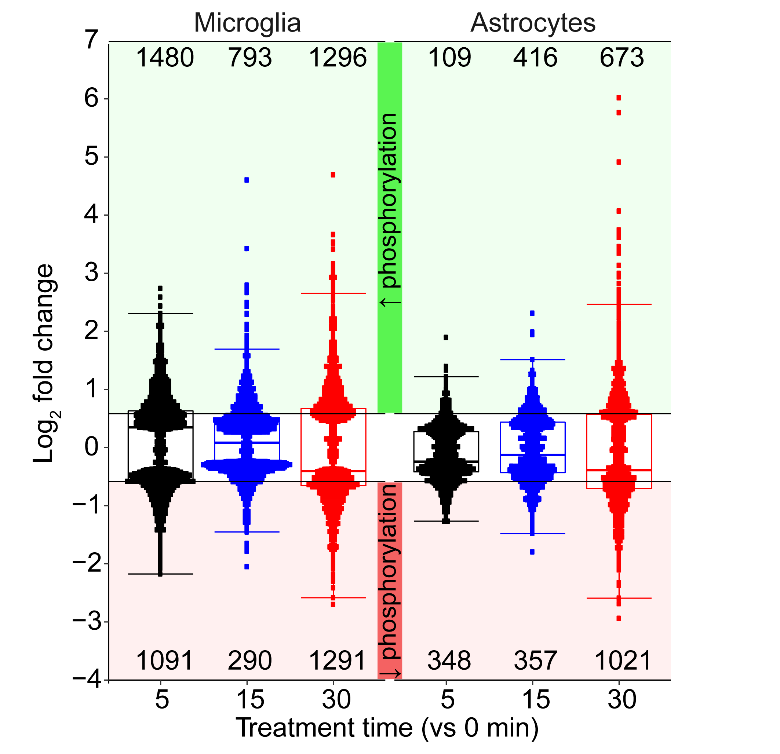
**

**Figure S5. Significantly regulated phosphosites after IFN-α-treatment in microglia and astrocytes.** Box and dot plot of significantly altered phosphosites revealed a distinct pattern of regulation across cell types. Numbers of significantly altered phosphosites are given for each time point.

**
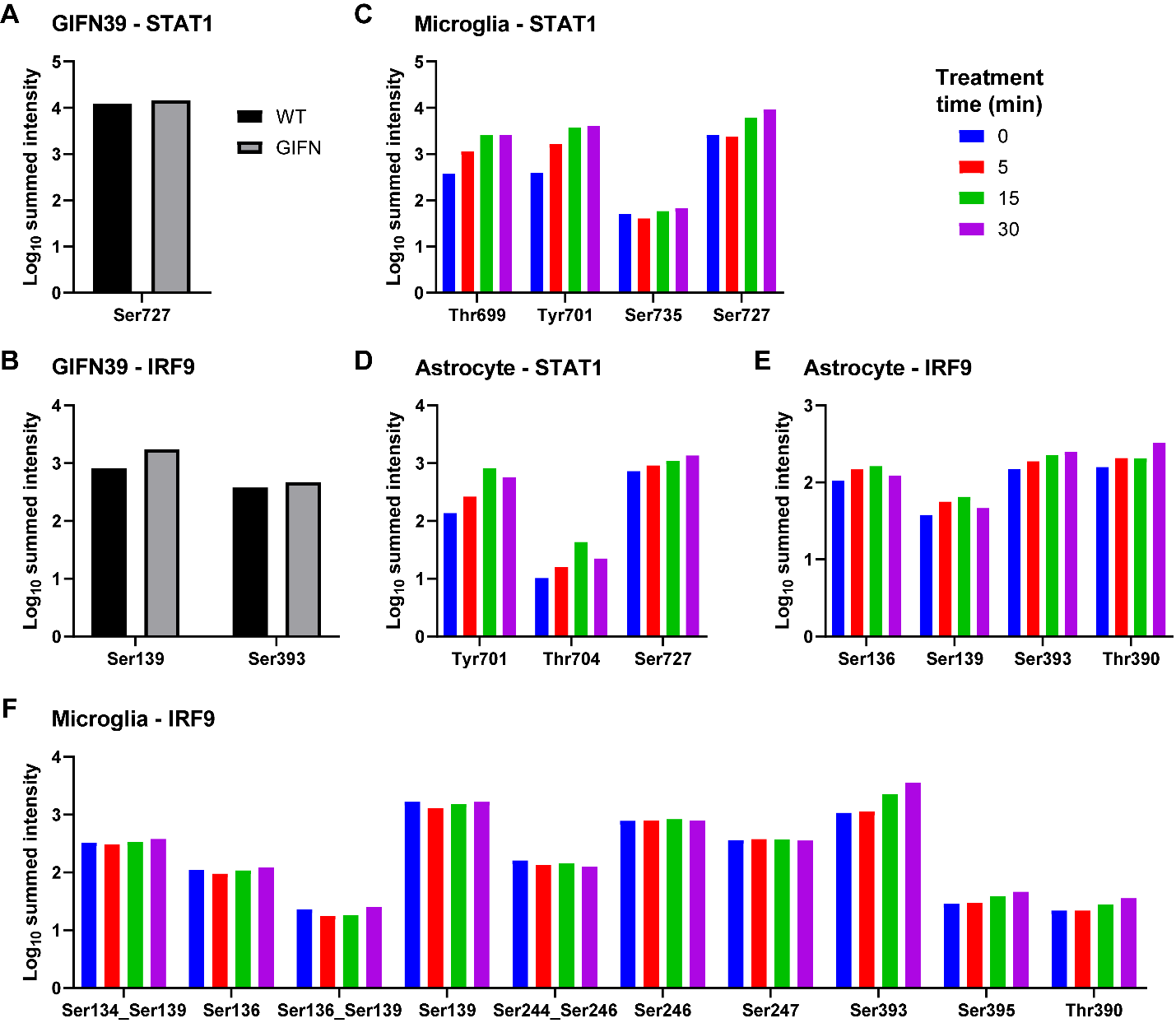
**

**Figure S6. Summed intensities of detected phosphosites on STAT1 and IRF9.** Abundance of phosphorylated sites (log_10_ summed intensity) on STAT1 and IRF9 detected in (A-B) WT and GIFN39 mice and (C-F) IFN-α-treated microglia and astrocytes for 0, 5, 15 and 30 min.

**
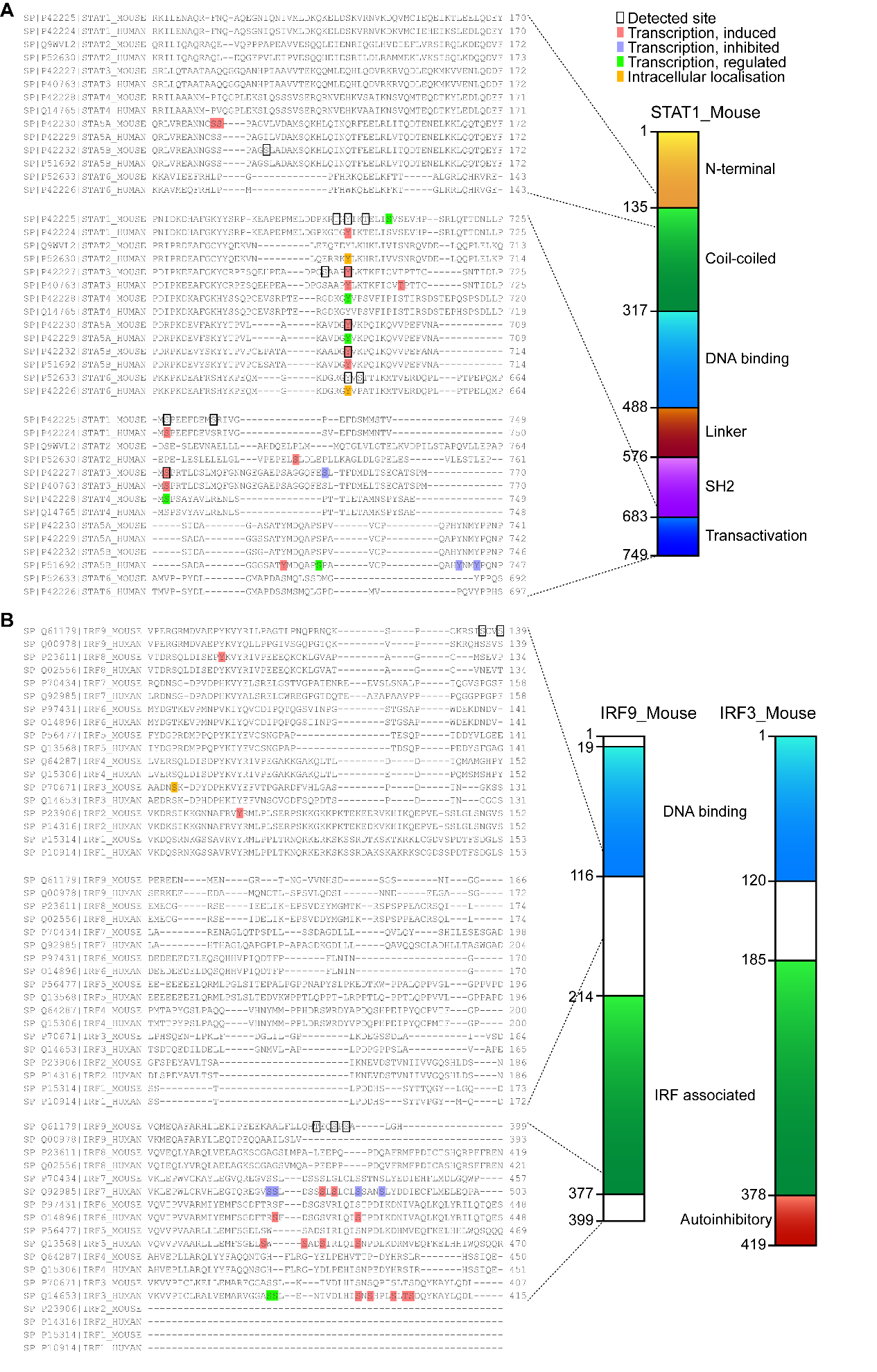
**

**Figure S7. Annotated sequence alignment of STAT and IRF proteins with detected IFN-α-regulated phosphosites.** Aligned mouse and human protein sequences of (A) STATs and (B) IRFs with annotated phosphosite function based on information from PhosphoSitePlus, with a focus on their effect on transcription. Regions of interest are shown against protein domains of the respective proteins. IRF3 was included as a representative of IRF1-8.


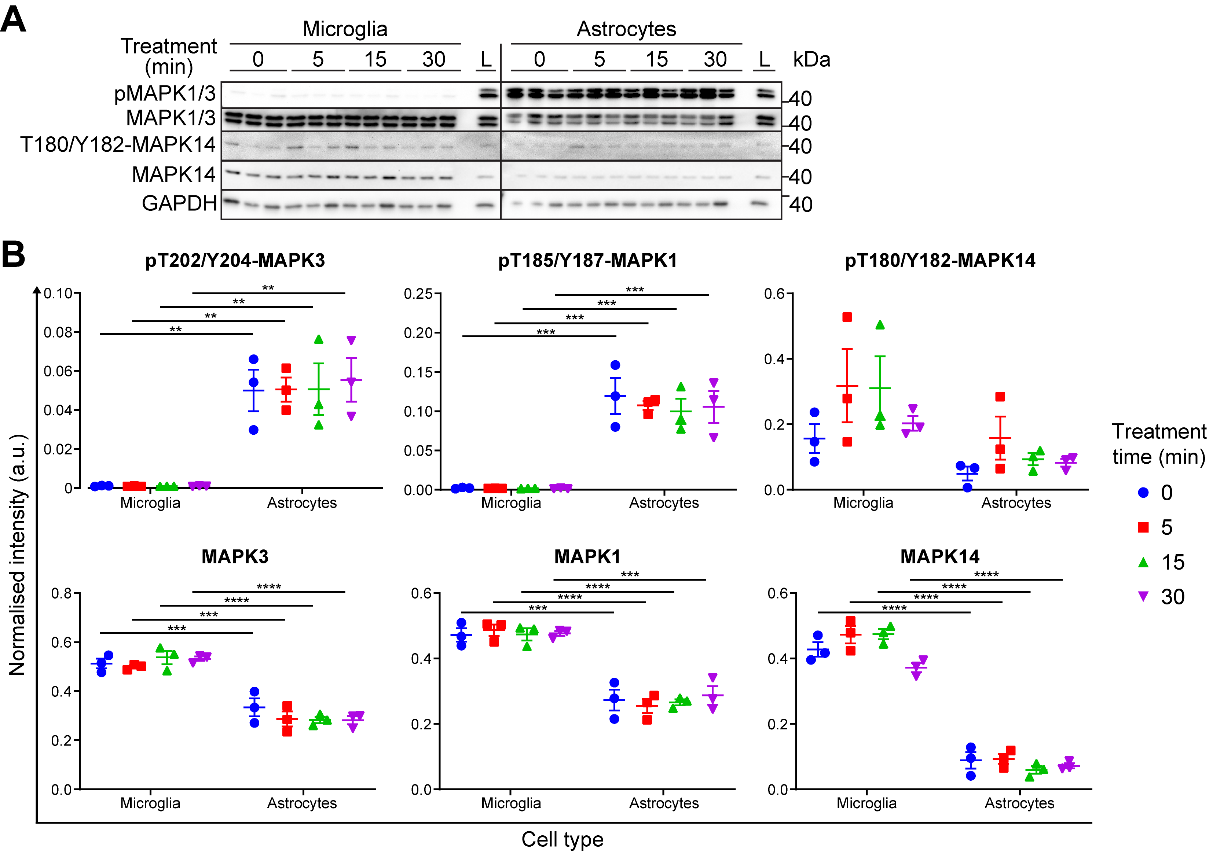


**Figure S8. Immunoblots for changes in activation of a subset of MAPK members in IFN-α-treated microglia and astrocytes.** (A) Immunoblot of whole protein lysates from IFN-α-treated microglia and astrocytes (n = 3 per cell type per time point). Sample “L” was a cross-membrane loading control of a pooled protein from all 30 min IFN-α-treated microglia and astrocyte samples. GAPDH is the same as shown in Fig. 6B. (B) Densitometric quantifications of immunoblots. Mean ± SEM are shown. *P < 0.05, **P < 0.01, ***P < 0.001 and ****P < 0.0001 compared to the respective 0 min of the cell type or between indicated samples as determined by two-way ANOVA with Tukey’s post-test.
