# Supplementary figures and images for: A novel phosphoproteomic landscape evoked in response to type I interferon in the brain and in glial cells
Source: J Neuroinflammation. 2021 Oct 16;18:237. doi: 10.1186/s12974-021-02277-x (PMC8520650; doi:10.1186/s12974-021-02277-x)

Source data for Fig. 6A and Fig. S7


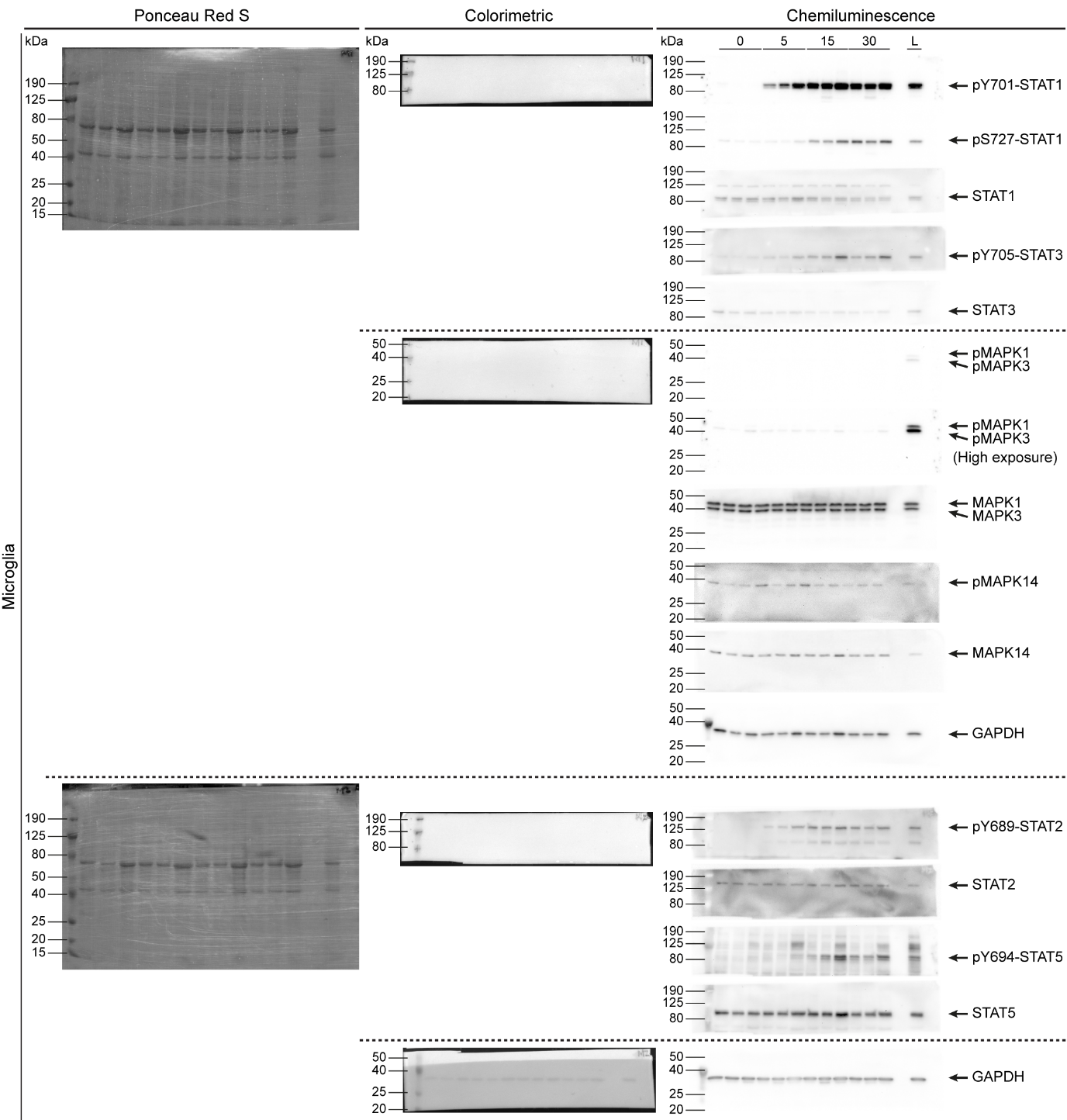


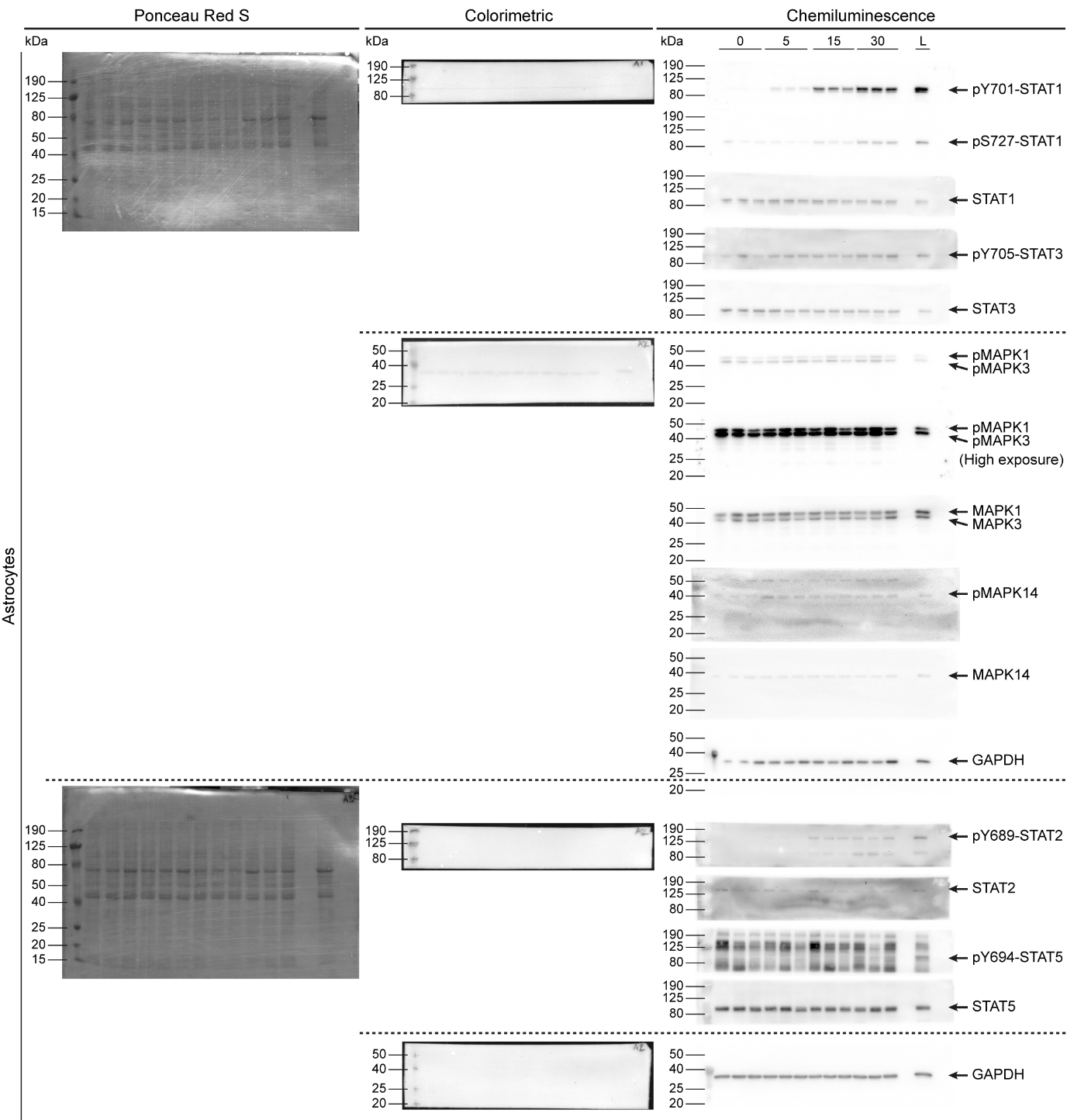


Source data for Fig. S2


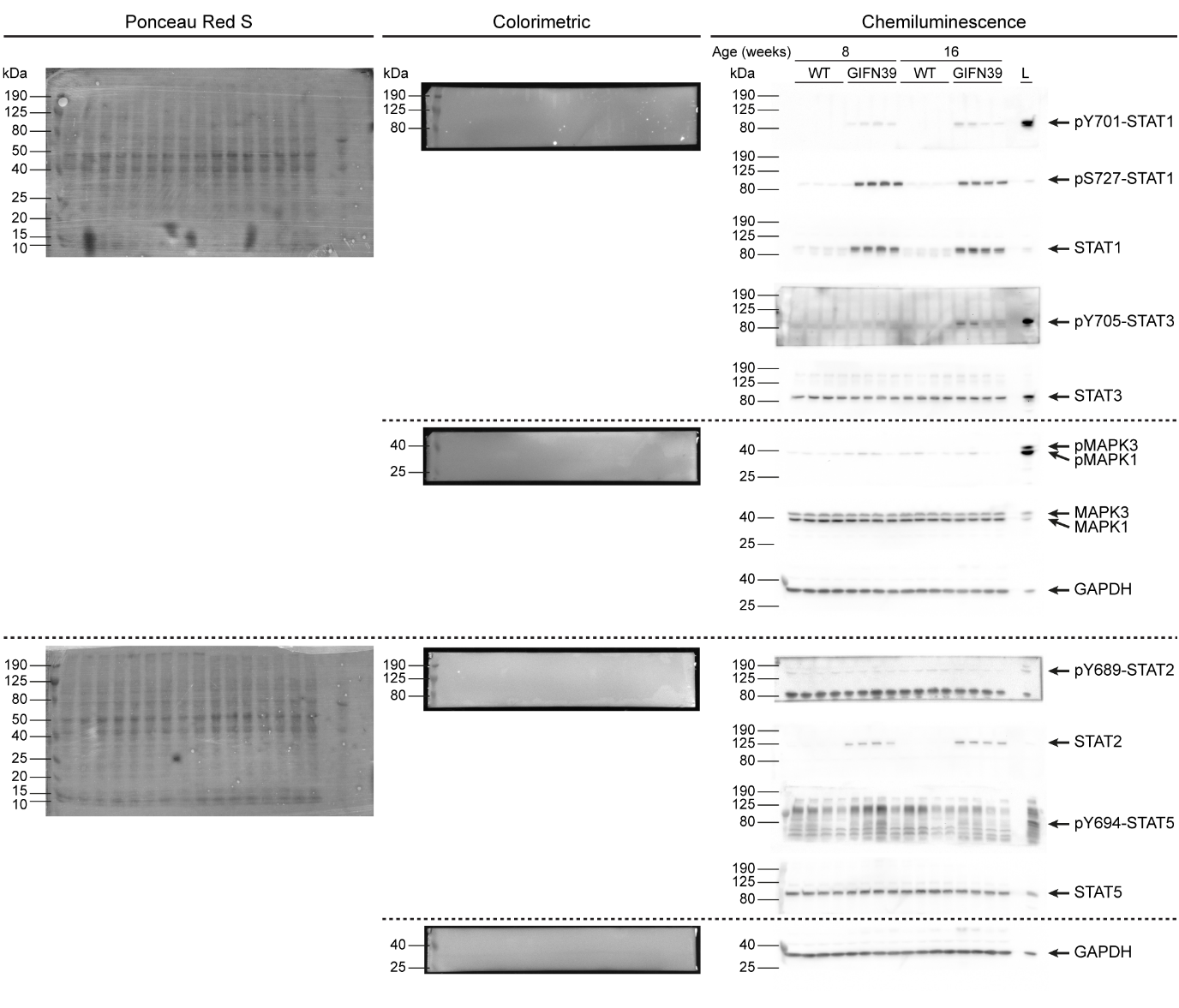


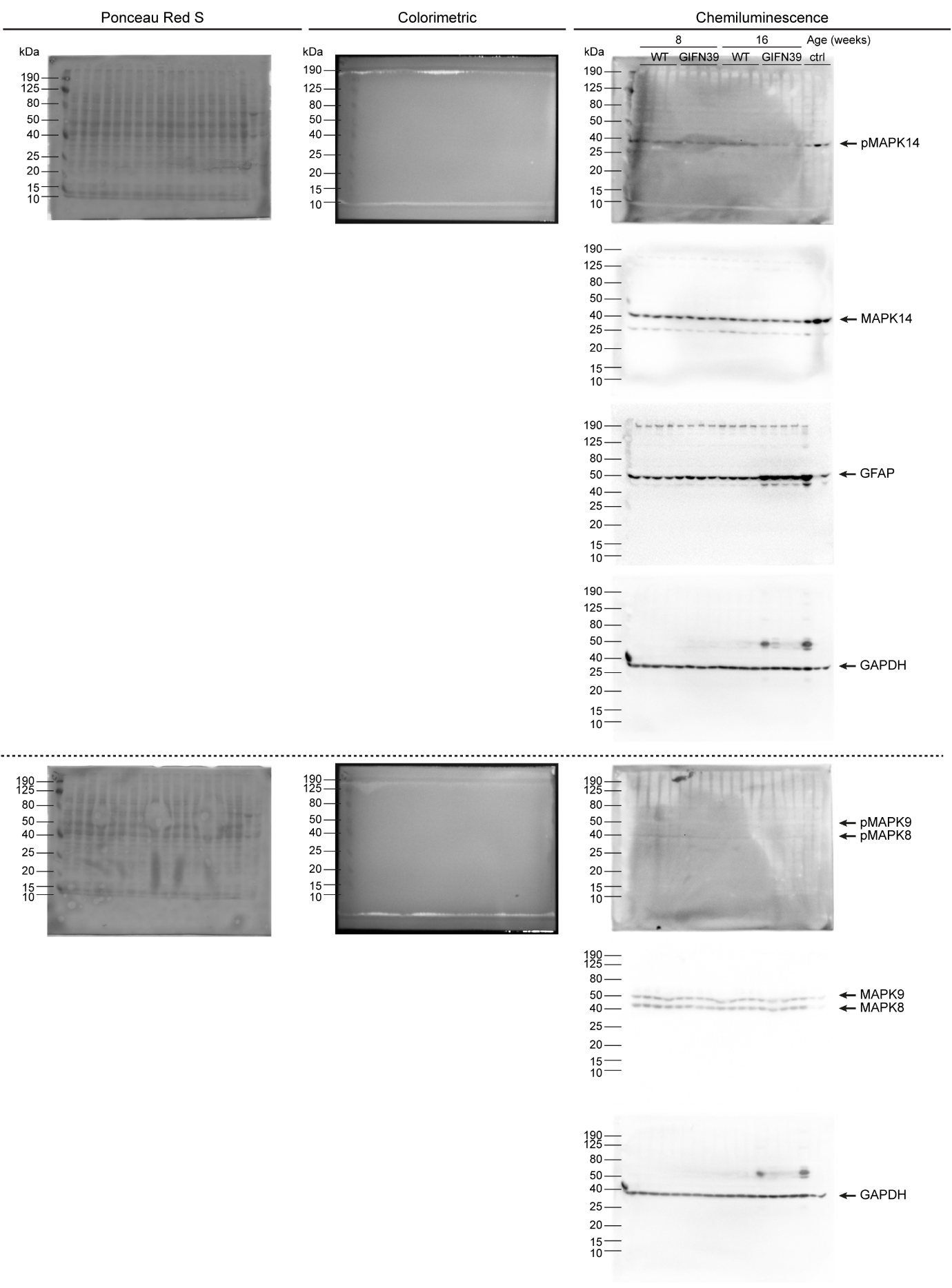


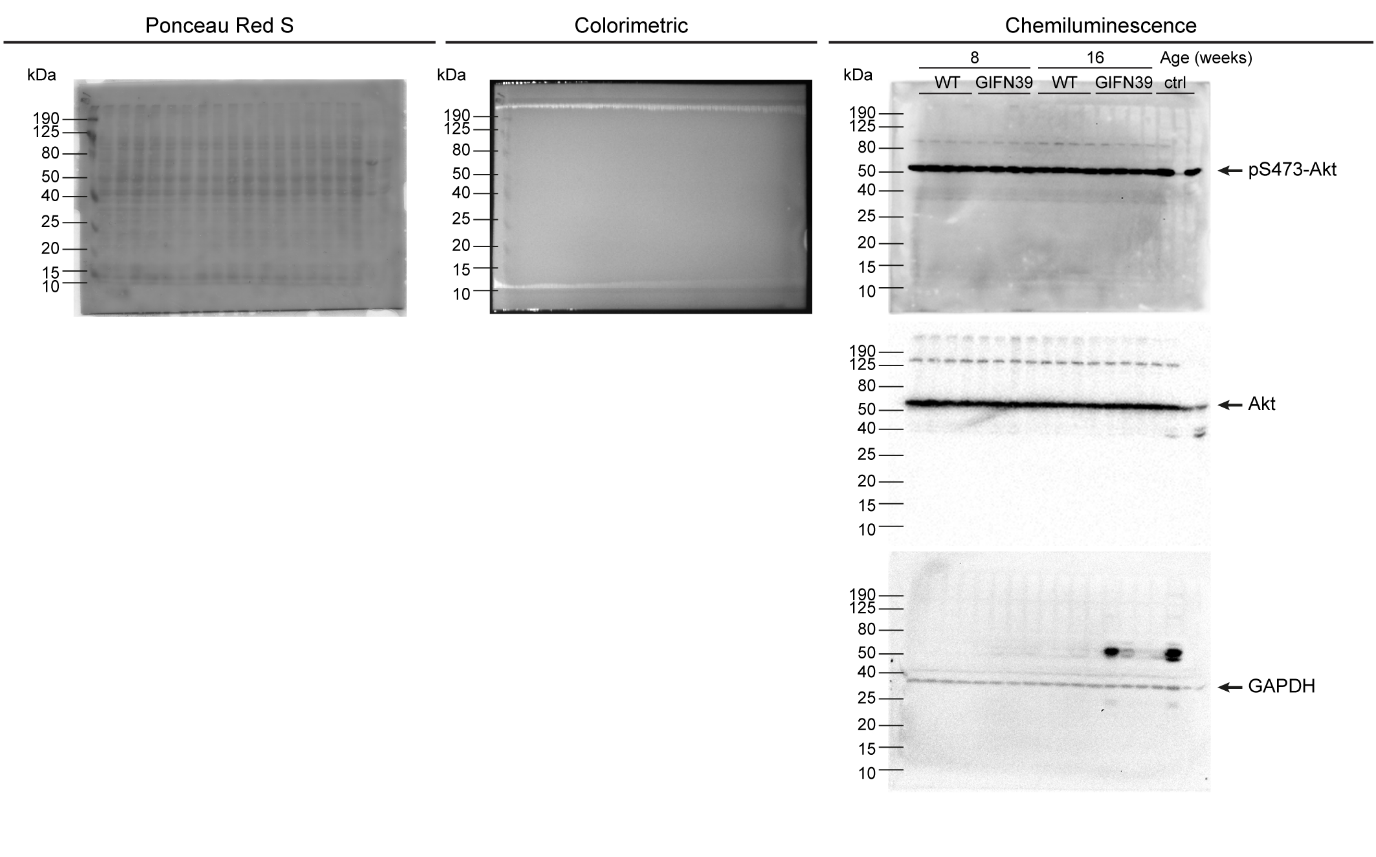

Supplement: Supplementary file 2 — Additional file 2. Source data. [file 12974_2021_2277_MOESM2_ESM.docx]
